# Placebo analgesia persistence: evaluating behavioural stability in repeated trials

**DOI:** 10.1038/s42003-026-10377-8

**Published:** 2026-05-28

**Authors:** Lewis S. Crawford, Damien C. Boorman, Ashleigh H. Wake, Allan Peng, Rebecca V. Robertson, James W. M. Kang, Furby Robson, Paul M. Macey, Kevin A. Keay, Luke A. Henderson

**Affiliations:** 1 School of Medical Sciences (Neuroscience), University of Sydney, Camperdown, NSW Australia; 2 Brain and Mind Centre, University of Sydney, Camperdown, NSW Australia; 3 School of Nursing and Brain Research Institute, University of California, Los Angeles, CA USA

**Keywords:** Sensory processing, Pain

## Abstract

Placebo analgesia is widely believed to rely on psychological, genetic, and biological factors. Whilst these factors have been assessed in single expressions of this phenomenon, few have documented its consistency over time, limiting understanding of its stability, and by extension, consideration in non-experimental settings. Here we show in 30 control participants by combining magnetic resonance imaging and a longitudinal design spanning five sessions, the psychological and neural substrates underpinning Placebo Analgesia response consistency.

## Abstract (web summary)

Longitudinal response conditioning to a sham analgesic alongside psychological and brain imaging evaluation revealed the behavioural consistency of Placebo Analgesia and associated psychobiological markers in pain-free humans.

## Introduction

Placebo analgesia is the powerful phenomenon in which pain inhibition is evoked by an inert substance or contextual cues via positive expectations^1^, conditioning effects^2^, or environmental associations^3–5^. Not all individuals express placebo analgesic responses, with most studies reporting placebo responses in approximately 35–67% of pain-free individuals and slightly lower rates in individuals with chronic pain^6,7^. Remarkably, a large meta-analysis on osteoarthritis estimated that placebo effects account for approximately 75% of the analgesic responses to commonly used medications^8^, and other studies estimate the effects to be in the 50–75% range^9^. Given the significant economic and methodological investment in accounting for placebo effects in almost all clinical trials, it would be extremely valuable if it were possible to not only predict whether an individual will display a placebo response or not, but more importantly, whether this response is consistent across timeframes typically used in clinical trials.

Dozens of studies have attempted to identify the factors that predict an individual’s likelihood of exhibiting placebo analgesia and the strength of their response. These factors include demographics^10^, genetics^11^, personality traits^12^ and contextual influences^13^. For instance, it has been reported that individuals who are (i) more trusting of the healthcare provider administering the placebo, (ii) more open to novel experiences, and (iii) reward-seeking tend to exhibit stronger placebo analgesia. Additionally, certain gene variants linked to dopaminergic and cannabinoid systems have been shown to influence placebo responding^11^. However, these studies all report relatively weak associations between these variables and placebo analgesic responses, and none of these factors provides sufficiently strong predictive value for clinical or clinical trial settings. This raises the critical issue that assessing placebo responses at a single time point may not accurately predict an individual’s future responses. In fact, this may explain why the associations observed across studies are often weak—individuals may exhibit placebo analgesia in some situations but not in others. Surprisingly, only a handful of studies have examined whether an individual will consistently demonstrate placebo analgesia to the same placebo administration over time.

The most influential study exploring placebo consistency found that using pharmacological conditioning in postsurgical pain patients, one could not accurately predict subsequent placebo responses based on the initial placebo response^14^. Another study reported that placebo responses were correlated across two experimental sessions when the same experimental paradigms were used in pain-free individuals^15^. Whilst these studies have attempted to explore placebo response consistency, methodological limitations such as the limited number of repetitions and the use of individuals with chronic pain mean that whether one can identify an individual that will display a consistent placebo response remains unresolved. Indeed, for the purposes of clinical trial selection, it would be particularly beneficial if one could predict and select only individuals that would consistently not experience placebo-induced analgesia to better detect the treatment effect.

Recent brain imaging studies have revealed that placebo analgesia is associated with recruitment of descending projections from the dorsolateral prefrontal (dlPFC) and cingulate cortices to the midbrain periaqueductal gray matter (PAG), which in turn modulates incoming noxious inputs at the level of the dorsal horn via the rostral ventromedial medulla (RVM)^4,16–20^. Of these cortical nodes, the dlPFC has received particular attention in recent years, commonly activated through response conditioning of placebo analgesia using sham substances or through social observation^16,17,21,22^, and flexibly altering placebo analgesia expression when subjected to non-invasive brain stimulation^23,24^. Moreover, directed connectivity analyses leveraging functional brain imaging data have shown both direct projections from the dlPFC and via relay with the anterior cingulate as the predominant cortical-brainstem pathway associated with stronger placebo analgesia responses^18,25^. Importantly, the brainstem circuitry recruited by these cortical circuits is thought to be involved in mediating the effects of most analgesic drugs, including opiates^26^ and cannabinoids^27^. Consistent with this circuitry, a study comparing two clinical trials in individuals with osteoarthritis found that dlPFC functional connectivity predicts placebo responses^28^ and two follow-up studies showed that dlPFC-precentral gyrus functional connectivity predicted placebo responses in two different chronic low back cohorts and osteoarthritis pain patients^29^. In addition to these reported brain activity measures, the ability to classify individuals that display consistent placebo analgesia responses or non-responses could aid in fundamentally altering the way in which we structure clinical trials and greatly improve our capacity to determine novel analgesic medication effectiveness and associated neural circuitry involved.

Several critical issues remain unanswered: (i) given the same experimental conditions, do some individuals display a consistent placebo response over time, (ii) are consistent or varying placebo responses associated with a specific psychological profile and (iii) what are the brain circuits that underpin consistent or varying placebo responses. If consistent placebo responders or non-responders exist and the neural circuitry underpinning these responses are defined, we will be able to improve clinical trial methodology and outcomes by preselecting individuals that are unlikely to display placebo effects and by assessing the true underlying circuitry responsible for an analgesic treatment effect. The aim of the present study was therefore to determine whether pain-free, healthy individuals show a consistent placebo analgesic response across a time period typically used in clinical trials (~8 weeks), and whether this stability (or instability) relates to a specific psychological profile and/or neural activation patterns. We hypothesised that individuals would follow similar analgesic response patterns across subsequent sessions, and that these responses would be driven by changes in neural activity consistent with brain regions associated with single-trial expression of placebo analgesia, such as the dlPFC and anterior cingulate cortices. Further, we hypothesised that a combination of psychological factors and patterns of brain activity could strongly predict the consistency of placebo responses.

**Fig. 1:**
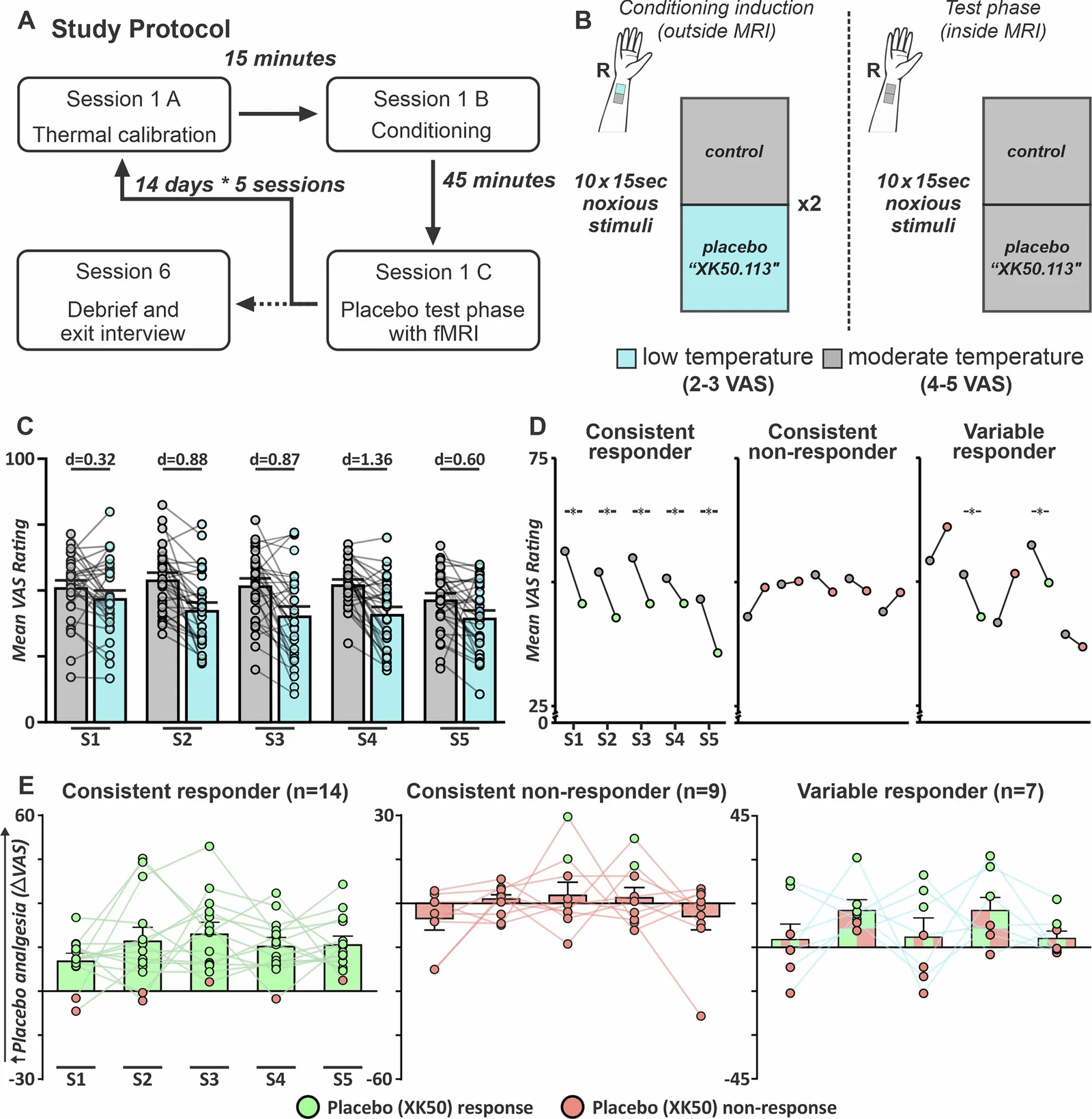
Methodology, placebo analgesia induction and response profiles over time. **A** *Study protocol*. At study commencement, participants (*n* = 30) completed a questionnaire battery and received a false information statement describing the qualities of XK50.113 (in reality, a placebo) in relieving pain. Each participant then undertook five experimental conditioning and test sessions, each separated by approximately 14 days. After five sessions had been completed, participants were debriefed as to the true methodology and conducted a one-on-one exit interview with the experimenter to establish discretion and ensure they had not realised the true study methodology before its conclusion. **B** *Conditioning procedure*. A standard response conditioning protocol was used to induce placebo analgesia to acute thermal noxious stimuli. Calibration of temperatures which would elicit a low- and moderate-pain response were calculated each session. Participants were informed that both the control and the XK50.113 cream would be receiving identical intensity stimuli throughout this phase; however, we deceptively reduced the temperature applied to the placebo cream to invoke expectations and reinforce beliefs of the placebo cream’s effectiveness. Then, during each visit, whilst inside the MRI scanner, both creams received identical intensity stimuli to produce the placebo analgesia phenomenon. **C** *Main effects analysis of pain intensity differences between cream sites*. Gray and blue bars indicate the mean pain intensity recorded at each cream site during each session across all 30 participants in the cohort. Individual datapoints are represented by circles overlapping each bar, with lines connecting each participant's control and placebo pain response during each session. Error bars indicate the standard error of the mean, and printed values represent repeated effect size Cohen’s *d* values. **D** *Group assignment*. Permutation testing was performed using each participant’s test phase pain intensity ratings during each experimental session (10,000 bootstrapped samples, random sampling with replacement) to identify how many significant placebo analgesic responses individual participants demonstrated across the five experimental sessions. Three groups emerged: consistent responders, consistent non-responders, and variable responders. A sample response pattern is shown for each of these three groups. **E** *Individual test VAS data*. Of the total sample, 14 participants (46.67%) were assigned as consistent placebo responders, 9 as consistent non-responders (30%), and 7 as variable responders (23.33%). In line with their assigned groups, in consistent responders, we observed significant reductions in pain intensity expressed on the placebo relative to control cream sites in four or more experimental sessions. In consistent non-responders, one or fewer placebo analgesia responses were identified, and variable responders expressed two or three placebo analgesia responses. Bars indicate the mean (±SEM) pain intensity change between cream sites during each session within each group, with a positive value indicating a greater placebo analgesia response (i.e. less pain perceived on the XK50.113 placebo relative to control cream). Individual participant datapoints across time are represented by line-connected circles. Green bars and circles represent a significant placebo analgesia response, and red bars and circles a non-response. MRI magnetic resonance imaging, fMRI functional magnetic resonance imaging, VAS visual analogue scale, S1–S5 sessions one to five.

In this work we analyze 30 participants (18 females; mean ± SD age 21.7 ± 2.5), measured longitudinally across five experimental sessions each separated by 2 weeks, implementing a response conditioning placebo analgesia paradigm, recording psychological measures prior to study commencement, and measuring functional magnetic resonance imaging (fMRI) brain activation patterns during each of the five experimental sessions (Fig. 1A). During each session, through surreptitious application of lower-intensity noxious thermal stimuli to a forearm site where a placebo cream was applied relative to an adjacent control cream, we conditioned participants to believe the placebo cream was acting to reduce thermal pain. The placebo cream was in fact identical to the control cream and with no analgesic properties (labelled 'XK50.113'; a novel topical anaesthetic specifically acting on heat pain). In a subsequent test session, whilst collecting fMRI scans, we applied a series of identical stimulus intensities to both cream sites ('moisturiser'/control; 'XK50.113'/placebo) and recorded each participants’ perceived pain intensity in real time (Fig. 1B). After each participant had completed all five experimental sessions, they were debriefed to the true nature of the methodology and necessary deception.

## Results

### Participants stratify into consistent placebo responders, consistent placebo non-responders, and variable placebo responders across five experimental sessions

Repeated effect size analyses (Cohen’s *d*) employed across all participants average VAS ratings to each of the control or XK50.113 placebo creams revealed a mix of small to very large effect sizes (range = 0.32–1.36) (Fig. 1C). Two-way repeated measures ANOVA revealed a main effect of cream site application *F*[1, 29] = 32.16, *p* < 0.001, and an interaction of cream × time such that across all participants, placebo responses differed between sessions *F*[4, 116] = 2.68, *p* = 0.03. No significant main effect of time was identified, *F*[4, 116] = 2.19, *p* = 0.07. Next, each participant’s average VAS ratings for each session were sequentially entered into a permutation model (10,000 bootstrapped samples with replacement), i.e. pain intensity rating during stimulation on the *control cream* versus pain intensity ratings during noxious stimulation on the *placebo cream*, for each of the 5 test sessions in each individual. Permutation testing then characterised each individual’s pain intensity change at each session as either a placebo *response* or a placebo *non-response*. Three clear response patterns emerged: group 1: participants displayed an analgesia in four or more experimental sessions—'*consistent responder'*; *n* = 14 (46.67%); group 2: participants displayed no analgesic responses in four or more experimental sessions—*'consistent non-responder'*; *n *= 9 (30%), and group 3: participants displayed inconsistent placebo analgesia responses—*'variable responder'*; *n* = 7 (23.33%) (Fig. 1D and Supplementary Table 1). Individual participant response patterns in each of these three groups is shown in Fig. 1E. Single factor ANOVA revealed that these groups did not differ in either conditioning or test stimuli temperature, or in average control site pain intensities (session 1: *F*(2, 27) = 2.49, *p* = 0.10; session 2: *F*(2, 27) = 1.19, *p* = 0.32; session 3: *F*(2, 27) = 0.70, *p* = 0.50; session 4: *F*(2, 27) = 3.20, *p* = 0.06; session 5: *F*(2, 27) = 0.22, *p* = 0.81), suggesting that these response patterns were not driven by differential thermal sensitivities (Supplementary Tables 2 and 3). Moreover, no between-group differences were identified with respect to age or sex identified at birth (Supplementary Table 4). One participant reported a history with chronic pain, which at the time of study completion had resolved, who was classified as a variable responder. The remaining 29 participants reported no history with or were currently experiencing chronic pain. In only two participants did we observe a significant linear relationship between pain intensity change and session number, suggesting habituation and learning effects over time did not drive the delineation between groups (Supplementary Fig. 1).

### Response consistency patterns are better predicted by the initial response than the psychological profile

**Fig. 2:**
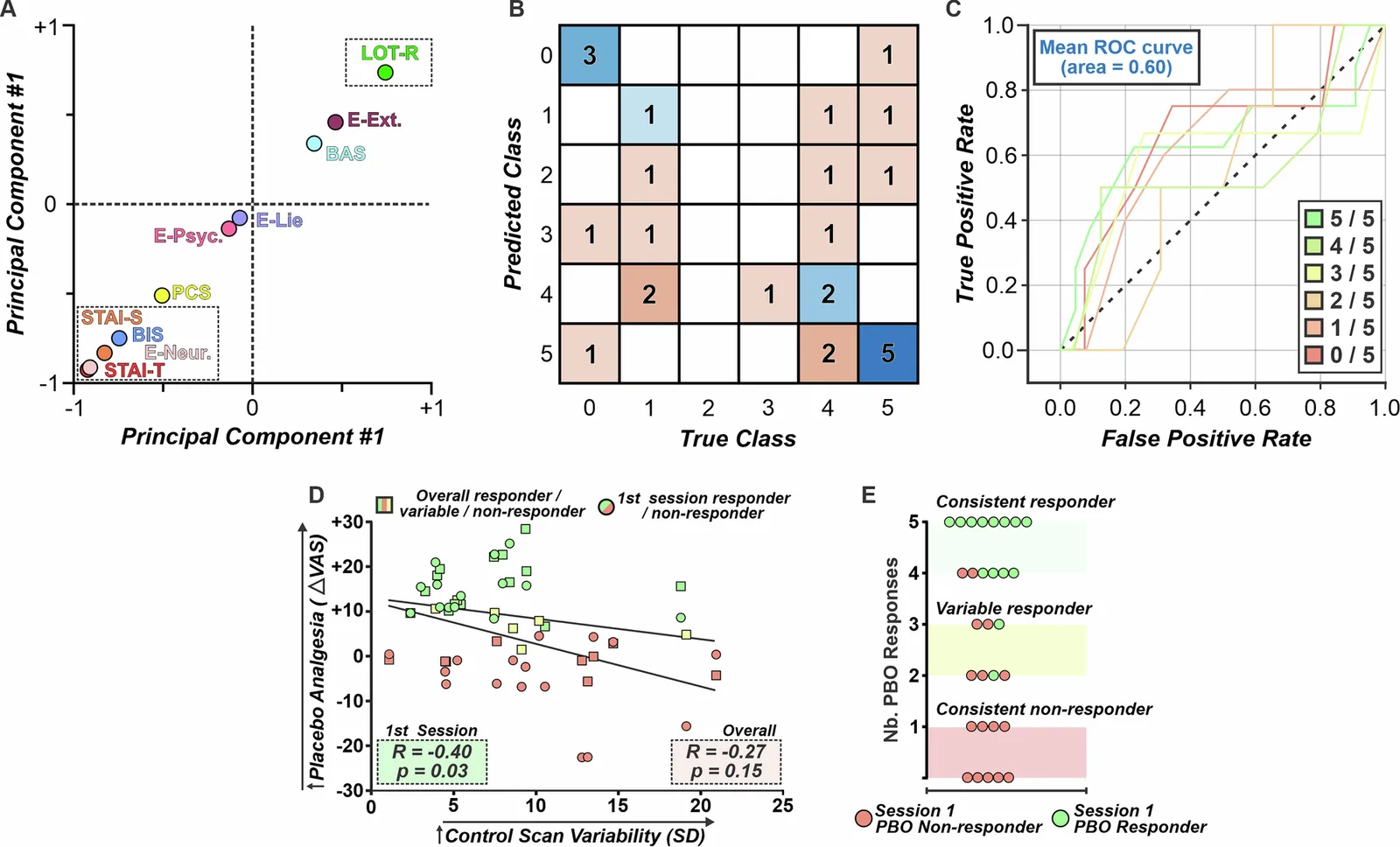
Defining the psychosocial composition of placebo response consistency. **A** *Principal component regression factor reduction*. Each participant’s responses to the administered questionnaires (*n* = 30) were entered with the outcome variable of interest, the number of times evoking a significant placebo analgesia response. Principle component analysis was then conducted to determine the questionnaires which contributed most strongly to the first principal component, which explained 59.10% variance. This indicated that both the state- and trait components of the state-trait anxiety inventory, the neuroticism sub-score of the Eysenck personality questionnaire, the behavioural inhibition sub-score of the behavioural inhibition and avoidance scales, and the life orientation test-revised questionnaire of trait optimism were significantly related to placebo analgesia response ratio within our 30-participant sample. **B** *Decision tree model analysis to determine predictive accuracy.* Within the classification learner toolbox of MATLAB version 2023a, each participant’s scores on the questionnaires, reduced by PCR and their response ratio out of 5 were entered into a medium decision tree model. This model returned an accuracy score of 36.7%, with notably superior accuracy in determining consistent placebo responders and non-responders— that is, those who evoked significant placebo analgesia in four or more or one or fewer out of the five experimental sessions. **C** *True/False positive rate receiver operating characteristic curve of the optimal classification model*. An alternate visualisation of the optimal efficient logistic regression model achieved an average area under the curve score of 0.60, indicating that the model comprising the reduced feature set of psychological questionnaire scores was neither accurate nor selective in delineating consistent placebo analgesia responders. Despite this, AUC for 5/5 and 0/5 responses were 0.66 and 0.65, demonstrating relatively greater accuracy in determining participants within these classes rather than variable placebo responders. **D** *Baseline pain perceptual variability predictive capacity of subsequent placebo responses*. Since it was previously shown that variability of pain intensity during conditioning on a control site, linear regression was performed to determine whether this association was predictive of longitudinal placebo responses from the initial session 1 conditioning round. A significant inverse association between session 1 control pain perception variability and subsequent placebo responses was identified, however this association was abolished when each participant’s overall mean placebo responsivity across all five sessions was entered. That is, more consistent pain behaviours in the initial session predicted initial, but not overall, placebo analgesia responses. **E** *Initial placebo responses predictive capacity of longitudinal placebo responses*. The classification of each participant as a placebo responder or non-responder in session 1 was compared with their overall final classification as either a consistent placebo responder, consistent placebo non-responder, or variable responder. This revealed that 12/14 first session responders concluded the experiment classified as consistent placebo responders. Similarly, 9/9 consistent placebo non-responders were classified as placebo non-responders to their initial session. As an accuracy score, 21/30 (70%) of participants final group classification could be predicted by their initial classification in the first experimental session. LOT-R life orientation test-revised, E-Ext Eysenck [extraversion], BAS behavioural avoidance subscale, E-Psyc Eysenck [psychoticism], PCS pain catastrophizing scale, E-Neur Eysenck [neuroticism], BIS behavioural inhibition subscale, STAI-S state trait anxiety inventory [state], STAI-T state trait anxiety inventory [trait], ROC receiver operating characteristic.

We first sought to determine whether the consistency of placebo responses was delineable via psychological measures. Direct comparison between groups using single-factor ANOVA revealed no significant differences between groups (Supplementary Table 5), and no significant linear relationships were identified between these scores or either overall placebo responsivity, or number of significant responses across sessions (Supplementary Table 6). We also determined whether a combination of our psychological measures could predict the likelihood of an individual mounting consistent placebo responses. To accomplish this, we first performed feature reduction by entering each participant’s questionnaire scores into a principal component regression analysis, which revealed a first principal component accounting for 59.67% variance in the number of placebo analgesia responses generated across the five experimental sessions. Specifically, questionnaires which contributed most strongly to this component were: the life orientation test-revised form, the behavioural inhibition scale, both the state and trait versions of the state/trait anxiety inventory, and the neuroticism subscale of the Eysenck personality questionnaire (EPQ) (Fig. 2A).

These values were next entered into the classification learner toolbox to determine how well these questionnaire scores performed in predicting an individual’s placebo response consistency. Feature selection was limited to only those questionnaires above that demonstrated a significant contribution to the first principal component, and five-fold cross-validation was employed to limit the effects of overfitting. Model training revealed that a medium decision tree approach combining scores on each of these five questionnaires was only capable of predicting the number of times our participants showed a significant placebo response with 36.7% accuracy. Notably, model performance was skewed such that 50% of consistent placebo responders were correctly classified by the model, however it was unable to predict any of the seven variable responders (Fig. 2B). Visualising this model as a receiver characteristic response curve returned an average area under the curve score across the six possible classes (zero to five out of five responses) of 0.60 (Fig. 2C). Overall, these data suggest that at least in our sample, neither a single nor combined score on common pain-related psychological questionnaires were capable of predicting the stability of placebo analgesia responses across time.

Since we have previously demonstrated a significant relationship between perceived pain intensity variability during conditioning and subsequent placebo responses, we next sought to determine if this relationship may instead hold predictive value in delineating consistent placebo responders and non-responders. Linear regression revealed that pain intensity variability on the control cream site in the first experimental session showed a similar relationship with placebo analgesia to that which we have shown previously. That is, those more consistent in their pain rating patterns produced a stronger placebo analgesia. However, this relationship was abolished when considering their overall placebo responsiveness across all five sessions (Fig. 2D).

Finally, we considered whether participants were more likely to retain or switch their group classification between experimental sessions across the longitudinal design of the experiment. That is, were participants initially classified as placebo responders more likely to go on to be consistent responders and vice versa. Of the 14 participants classified as consistent responders, 12 demonstrated a significant placebo analgesia in the first experimental session, with the remaining two satisfying the criteria to be variable responders (i.e. they expressed placebo analgesia in 2 or 3 of 5 experimental sessions). Likewise, all 9 consistent non-responders did not demonstrate placebo analgesia in the first session. In other words, if an individual did not display a placebo analgesia in the first session, they did not display a placebo analgesia for at least 3 of the subsequent 4 experimental sessions. Overall, our data shows that in 70% of participants (21 of 30), the first placebo response could predict their eventual classification as either a consistent placebo responder or non-responder (Fig. 2E).

### Repeated placebo exposures evoke functional activation changes in cognitive-evaluative and pain-modulatory brain regions

An initial main effect of pain analysis conducted in all participants over time revealed increases and decreases in blood oxygen level dependent (BOLD) signal consistent with the canonical pain perception and modulation system. That is, increases were isolated to the primary somatosensory cortices (S1) and frontoparietal structures, such as the dorsolateral prefrontal cortices (dlPFC), the middle (MFG) frontal gyri and the anterior insular (aIns) cortices. Dissimilarly, decreased BOLD signal was observed in default mode network structures, such as the medial prefrontal cortex (mPFC) and within the precuneus (Supplementary Fig. 2).

**Fig. 3:**
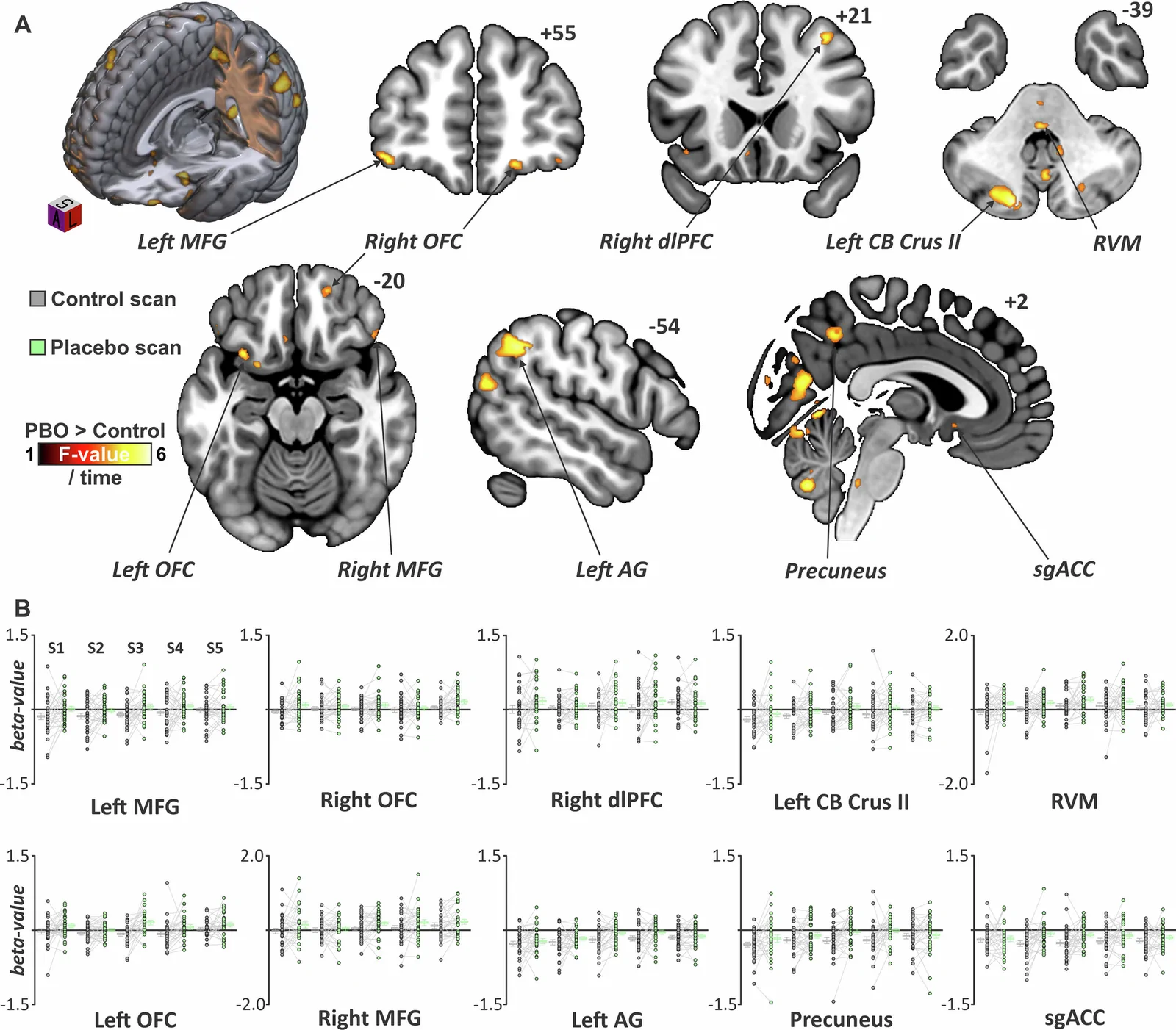
Altered placebo-related brain activity patterns over time. **A** *Main effect of placebo over time in all participants*. A 30 × 2 × 5 flexible factorial analysis including factors of 'participant', 'scan' and 'time', was constructed within SPM12 with average perceived pain per scan included as a regressor of interest (*n* = 30). Significant increases in activation were observed within the bilateral middle frontal and orbitofrontal cortices, as well as within the right dorsolateral prefrontal cortex, left angular gyrus and cerebellar crus II, and within the rostroventromedial medulla and subgenual cingulate cortices. **B** *Signal intensity change extracted per session as a main effect of placebo*. Extraction of signal intensity values in both the control (gray bars) and placebo (green bars) scans revealed that largely average signal intensity was at baseline during control pain processing, and increased during placebo-related pain encoding in each of the five experimental sessions. Notably, within the left angular gyrus, and both the precuneus and subgenual cingulate cortex, negative signal intensity was observed during control pain processing, which increased but remained negative during placebo-related pain encoding. Average session mean (±SEM) signal intensity during stimulation of the control (gray bar), and XK50.113 placebo (green bar) creams are provided with interconnected dots and lines delineating each participant's activity change over time. A navigation cube and rendered image is provided in the upper left corner to aid in visualisation of significantly activated regions, with MNI co-ordinates in the upper right corner of each slice in either coronal, axial, or sagittal orientations, respectively. MFG middle frontal gyrus, OFC orbitofrontal cortex, CB Crus II cerebellar crus layer two, RVM rostral ventromedial medulla, AG angular gyrus, sgACC subgenual anterior cingulate cortex.

Next, by considering changes in BOLD signal between the two functional series, a main effect of placebo analysis was conducted and revealed that across all participants, noxious stimulation of the XK50 placebo relative to the moisturiser control cream over time was associated with significantly altered blood-oxygen dependent signal within several cortical areas believed involved in cognition—particularly the cognitive-evaluation of noxious inputs (Fig. 3A). Extraction of beta-values from each resulting cluster revealed that largely, these regions demonstrated increases in signal, specifically bilaterally within layer 2 of the cerebellar crus; the MFG; the orbitofrontal cortices (OFC); the right MFG; the left angular gyrus (AG); the left posterior parietal cortex (PPC); and in the midline precuneus and cerebellar vermis. Additionally, increased signal was also observed in a collection of pain-modulatory brain regions, namely, within the bilateral dlPFC and within the midline middle (MCC) and subgenual anterior cingulate cortex (sgACC) and rostral ventromedial medulla (RVM). Of all regions arising from the main effect analysis, only three clusters demonstrated signal intensity decreases, all consistent with sensory-discriminative systems of pain perception. Specifically, reduced signal intensity during stimulation of the XK50 placebo relative to the moisturiser control cream was observed bilaterally within the S1, and in the left posterior insula cortex (pIns) (Fig. 3B and Supplementary Table 7).

Inspection of overall signal intensity changes across these brain regions revealed characteristics of significantly decreased signal during control pain processing, which increased yet either remained reduced or near baseline during placebo pain processing within the CB Crus; the right MFG; the sgACC; left MFG; left AG; and within the precuneus. Additionally, a pattern of signal intensity change indicating near baseline signal during control pain processing, which increased significantly above baseline during placebo pain processing, was observed within the RVM; the cerebellar vermis; the OFC; the right MFG; the MCC and within the dlPFC bilaterally; and in the left PPC (Supplementary Table 7). Within the left posterior insula and S1, signal during control pain processing was significantly increased, reducing to near baseline in placebo pain processing. This pattern differed from the right S1, where the signal was significantly increased during both functional series, yet was decreased during placebo relative to control pain processing (Supplementary Table 7).

### Consistent placebo analgesia responders and non-responders differentially engage cognitive-evaluative neural systems

**Fig. 4:**
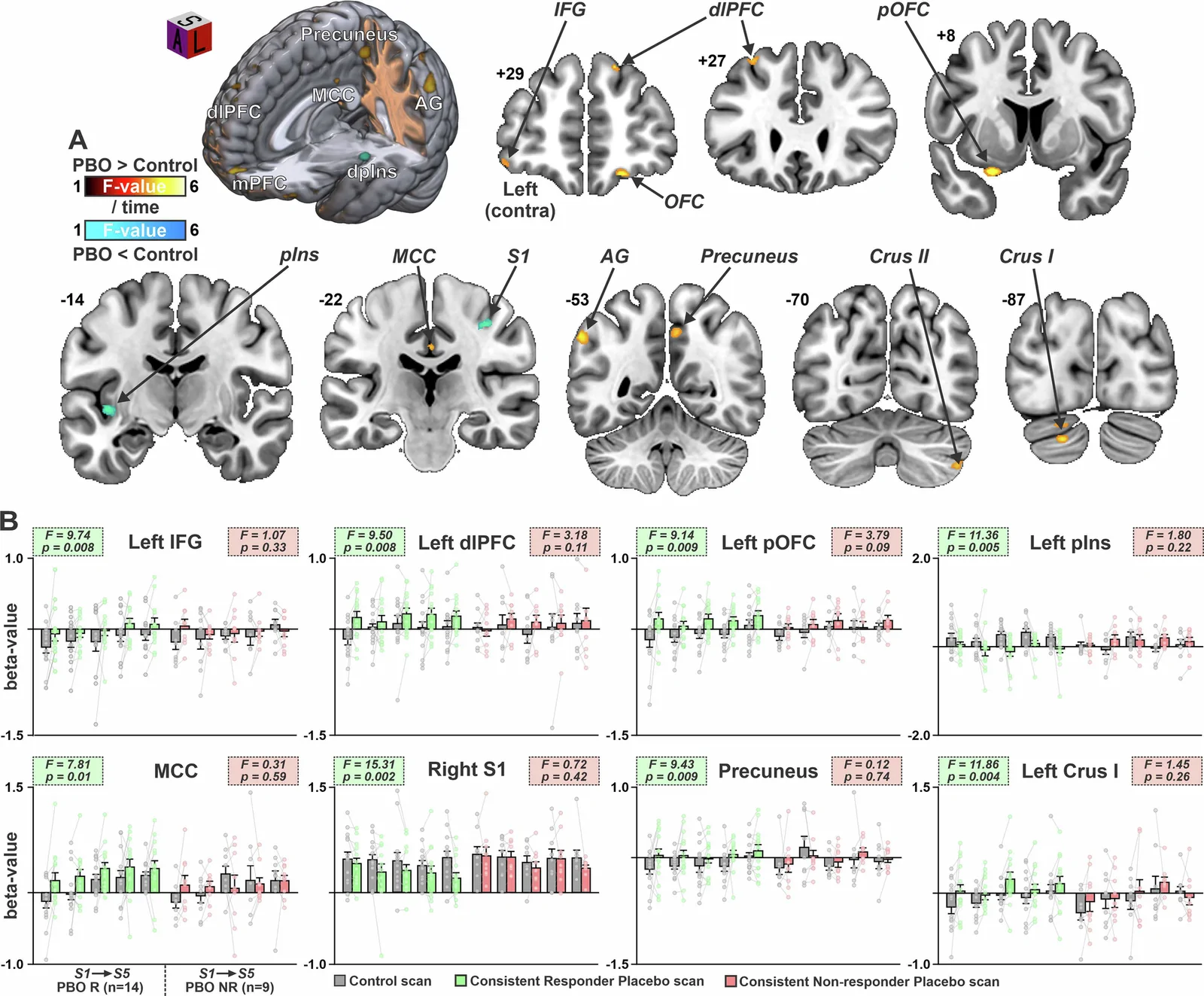
Masked consistent placebo responder main effect of placebo analysis. **A** *Cortical regions of altered activity during consistent and significant behavioural placebo analgesia*. A full factorial analysis conducted on the 14 classified consistent placebo analgesia responders with both 'scan' and 'time' loaded as separate two and five-layer factors, respectively, was conducted using the main effect of placebo contrast map of all participants as a volume of interest mask, thresholding analyses to only cortical regions altered in the context of a placebo administration. This revealed a subset of regions previously identified, which significantly increased in activity only in those participants who consistently mounted behavioural analgesia, consisting of the bilateral orbitofrontal and dorsolateral prefrontal cortices, the left inferior frontal and angular gyri, the left cerebellar crus I and the right crus II, and in the midline precuneus and middle cingulate cortex. Additionally, significantly reduced signal intensity during placebo relative to control cream noxious stimulation was identified within the left posterior insula and right primary somatosensory cortex. **B** *Signal intensity changes extracted within each consistent responder and non-responder group*. Resulting clusters of consistent responder main effect of placebo changes were saved as volume of interest masks and measures of signal intensity (beta-value) during each functional series extracted from consistent placebo responder and non-responder groups. Post hoc two-factor repeated ANOVA was conducted in each group separately, showing that these significant changes were isolated only to consistent placebo responder groups. Average session mean (±SEM) signal intensity during stimulation of the control (gray bars), and XK50.113 placebo (green and red bars) creams are provided with interconnected dots and lines delineating each participant's activity change over time. IFG inferior frontal gyrus, dlPFC dorsolateral prefrontal cortex, pOFC posterior orbitofrontal cortex, pIns posterior insula cortex, MCC middle cingulate cortex, S1 primary somatosensory cortex, AG angular gyrus, PBO R consistent placebo responder, PBO NR consistent placebo non-responder.

Next, to delineate whether the above neural changes were driven by or selectively altered within only those participants which consistently displayed a significant analgesic response to placebo exposure over time, a main effect of placebo analysis was conducted in only participants classified as consistent analgesia responders, using the above main effect changes as a binarized volume of interest mask. Surviving clusters were observed within most areas first identified in all participants, with increases in BOLD signal isolated to the bilateral CB Crus, MFG and dlPFC; the left OFC and AG; the right mPFC; and in the midline MCC and precuneus. Decreases in signal intensity were also observed specifically within the left pIns and right S1 (Fig. 4A and Supplementary Table 8). Extraction of beta values from both the consistent placebo responder and non-responder participants alongside post hoc two-way repeated ANOVA revealed that significant changes in signal intensity over time within each of these sites were only observed within those participants that consistently mounted a significant placebo analgesia response, and not in consistent placebo non-responders (Fig. 4B). Moreover, three-way repeated ANOVA was also conducted between all three groups, revealing a significant interaction effect of response class by site of stimulation (i.e. XK50 placebo or moisturiser control) specifically within the right mPFC, MFG and S1; and within the dlPFC bilaterally (Supplementary Table 8).

## Discussion

Our data shows that in the majority of individuals, placebo responsiveness is consistent over time and that 70% of individuals can be categorised as placebo analgesia responders or non-responders based on their initial response. Importantly, from a clinical trial design perspective, we found that all participants classed as placebo non-responders did not display a placebo analgesia in the first session. That is, one could reliably predict non-responders based on the first non-analgesic placebo response. Furthermore, we found that the consistency of placebo analgesia responses was not associated with any single nor combination of psychological factors, and while we provide further evidence for the predictive capacity of pain intensity variability for subsequent placebo analgesia, this marker is less reliable in predicting responses across time. Consistent with our hypothesis, we found that the consistency of placebo responses was associated with activity changes in higher order association cortices, such as the dorsolateral, orbitofrontal and cingulate cortices.

Several previous attempts have been made to determine the stability of the placebo phenomenon at the behavioural level. The most influential by Lasagna and colleagues in the 1950s used pharmacological conditioning in postsurgical pain patients and reported that one could predict no better than chance subsequent placebo responses based on an initial session^14^. However, more recently, this finding has been challenged with reports that the magnitude of placebo analgesia responses were significantly correlated across two experimental sessions up to 8 days apart^15^. Importantly, we used an experimental paradigm that allowed us to dichotomize individuals into responder and non-responder groups based on the variability and magnitude of pain intensity changes and then determine the consistency of this grouping over time. Importantly, while consistent placebo responders were defined solely based on pain reductions, a non-response encompassed both negligible changes in pain between the two cream sites, as well as paradoxical nocebo responses, or pain increases on the XK50.113 cream relative to control, despite this substance being delivered in the context of a placebo. We have demonstrated previously that such paradoxical reactions are present cross-sectionally in several behavioural endogenous analgesic mechanisms, including placebo analgesia^30^. While the focus of this investigation was to determine *analgesic response consistency*, it is relevant to observe that paradoxical nocebo responses continue to emerge in some individuals. That said, our data provide the first robust evidence that placebo responses are largely consistent over time when applying a consistent experimental paradigm, delivered in the same environment, and at least over a period of time that is similar to that used in clinical trials (i.e. 8 weeks).

Despite previous associations made between placebo analgesia and psychological traits, such as dispositional optimism and anxiety^12,31,32^, we found no such associations either between groups, as linear associations, or when combined with predictive modelling. In addition, previous efforts to identify a 'placebo personality' have reached a consensus that there exists no single personality trait that a priori identifies placebo responders, but rather certain psychological dispositions lend themselves to placebo responses in specific settings^31^. Whilst our questionnaire principal component regression could identify a component accounting for reasonable variance in the number of placebo analgesia responses across time, these scores could not predict with great accuracy the number of responses generated when subject to cross-validation under a decision tree style model. However, it was clear that of the questionnaires contributing to the first principal component were two clusters—a positive weighting on trait optimism and a negative weighting on a collection of questionnaires underlying emotional instability, anxiety and behavioural instability. Interestingly, both subscales of the state/trait anxiety inventory and the life orientation test revised form of dispositional optimism provided the strongest model contribution—measures which in the past have been either correlated with placebo response intensity^12^, or baseline pain thresholds^33^. While no significant differences were reported between our groups in these two measures—the graded difference in responses, such that consistent responders were highest in trait optimism and lowest in both state and trait measures of anxiety, and vice versa for consistent non-responders, reflects similar findings reported for test-retest reliability of placebo analgesia^34^.

Previously, we demonstrated that the amount of inherent variability a participant perceives to acute thermal pain during conditioning on a control site relates directly and inversely to subsequent placebo analgesia responses, and that this variability is encoded by activation and excitatory/inhibitory balance of the dlPFC^35^. In this experiment, we were interested to explore whether this association remained across time and whether pain rating consistency may act as a biomarker to placebo analgesia. Whilst an association was present specific to the first session—i.e. those more consistent in rating profile on the moisturiser control cream expressed greater initial placebo analgesia—this association was abolished when linear regression was performed using first session variability and overall mean placebo analgesia across all five sessions. This finding is echoed in preclinical literature, suggesting that during the acquisition phase of conditioned responses, variations to the conditioning or conditioned stimulus induce weaker conditioned behaviours^36,37^, and reinforces our previous finding that those participants who show greater pain intensity variability are less capable of forming strong stimulus-response associations required for the expression of conditioning-based placebo analgesia.

Our functional imaging results demonstrate that placebo administration and subsequent noxious stimulation of this placebo-treated site evokes consistent changes in brain activity within several higher-order cognitive-evaluative brain structures, such as the dorsolateral and orbitofrontal cortices. These areas were first identified across all participants, however further investigation of placebo response consistency demonstrated that significant changes were primarily driven by those participants that consistently mounted behavioural analgesia. The dlPFC has received considerable attention with respect to its role in placebo analgesia and is currently believed to be pivotal in mounting placebo analgesia. This is primarily due to changes observed in placebo expression when the dlPFC is subject to non-invasive brain stimulation^23,24^, as well as large-scale meta-analyses showing consistency in this region’s activation during placebo analgesia^38^. In addition to the dlPFC, this meta-analysis identified significant changes in brain function of the AG and middle cingulate resulting from placebo induction, areas also observed within our cohort and driven particularly by a greater placebo response consistency. These sites hold a role in cognition and integrating noxious inputs to contextual cues, such as in social observation and learning, and our findings likely reflect a broader cortical system being engaged, resulting from the conditioning process, leading to the cognitive modulation of perceived pain^22,39^.

We also recently showed in an ultra-high field fMRI study that the lateral/dorsolateral column of the PAG and its connectivity with the dlPFC and cingulate cortex is associated with placebo analgesia responsivity^18,35^. Our current findings extend these reports by revealing that consistent recruitment of the dlPFC, OFC and middle cingulate cortex relates directly to one’s ability to mount a consistent placebo analgesia. Indeed, whilst overall signal changes in the dlPFC and OFC were present, when parameter estimates were extracted in each group, we identified that significant functional changes in both these areas were isolated to consistent responder groups. OFC activation has been associated with pain reduction by reward and is believed to be a key neural hub in integrating cognition and affect with perceived pain^40^. Together, our findings may reflect that the OFC was activated during the relatively rewarding perception of pain relief by placebo administration, supporting that this site as necessary in determining the subjective value of reward^41,42^. We also found activity increases in the Crus II cerebellar cortex, a region that has recently been implicated in placebo analgesia and, in particular, its connections with the anterior cingulate cortex that are proposed to drive conditioned placebo analgesia in rodents^43^. Notably, in humans, Crus II displays functional connections with the dlPFC^44^, and has also been implicated in social cognition and behavioural selection^45^.

Moreover, in the bilateral dlPFC, IFG and left posterior insula cortex, we identified an interaction effect of group by cream site—indicating that activity in these areas significantly differed as a function of the resulting behavioural response. As above, the dlPFC is believed necessary for the generation of placebo analgesia, via its descending recruitment of pain-modulatory brainstem sites. Our results, however, suggest that alternate areas can also delineate these responses and align with a recent non-invasive brain imaging investigation where direct current stimulation to a cortical area consistent with our right IFG cluster enhanced placebo analgesia. In this same study, nocebo hyperalgesia was effectively inhibited via dlPFC stimulation, which related to functional connectivity changes between the dlPFC and the posterior insula^24^. It has long been upheld that behavioural modulation of perceived pain results from cognitive integration of prior knowledge with newly presented sensory information—that is, pain modulation can be modelled as a Bayesian system^46,47^. Our findings further support this ideology, with response classes being defined primarily through functional activation differences between consistent responders and non-responders in both cognitive-evaluative and sensory-discriminative cortical areas, such that placebo analgesia may result from an integration of each aspect of the pain neuromatrix^48^.

Our study is limited such that our findings and interpretation are bounded by a number of factors. We induced placebo analgesia via response conditioning, and several of the brain structures we identified have been closely linked to this method of conditioning. Whilst we cannot rule out signal changes that reflect generalised learning effects and stimulus-response relationships^49,50^, the brain regions we identified have been routinely tied with placebo analgesia elicited via both social observation and expectancy modulation—suggesting their importance in mounting the phenomenon regardless of method of induction^22,38,51^. Additionally, our sample consisted primarily of young adults that were pain-free. It is possible that placebo reactions in pain-free individuals differ to those in individuals with chronic pain, however placebo circuitry remains functional in individuals with chronic lower back pain, since chronic pain patients exhibit placebo response rates similar to those of pain-free controls. Moreover, dlPFC-connectivity strength predicts placebo responsivity during the placebo arm of clinical trials in individuals with chronic back pain and osteoarthritis, chronic pain^6,7,28,29^. In future studies, it would be of interest to determine whether consistency of placebo analgesia responses is conserved in those with chronic pain or in participants that have a history of resolved chronic pain. With respect to age, thermal sensitivity declines with age^52^, and we cannot assume that the same degree of consistency in the placebo analgesia phenomena would be conserved across the lifespan. Additionally, age and chronic pain have been shown to alter the anatomy of the same neural circuitry that we show underpin consistency of placebo analgesia (i.e. the dlPFC)^53–55^. Given that chronic pain and age do not appear to alter placebo responsivity rates, at least in single trial studies^56,57^, this suggests that the reported volumetric changes in these cohorts do not significantly alter placebo-related function, or that redundancy of neural function exists. Whether age or chronic pain effects placebo responsivity consistency over multiple sessions remains unknown and is an interesting direction for future research. That being said, in our hands and through our specific response conditioning design, we report that thermal sensitivity differences had no effect on placebo responsiveness.

Importantly, a feature of our experimental design is that each participant was assessed in the same environment (inside an MRI scanner) and using the same placebo paradigms during each experimental session. As such, our findings are constrained to only associating these consistency behaviours of placebo analgesia within a response conditioning model using a deceptively described sham topical ointment, and using short-lasting thermal noxious stimuli. Moreover, we cannot provide evidence for whether our cohort of consistent responders and non-responders would display these same behavioural patterns if the placebo substance were changed to a pill or in a social observation model, or if the type of stimulus was adjusted to a mechanical or visceral experimental model of pain processing. Future research leveraging the design we have now established, however, altering these experimental components during specific session numbers would be of benefit to improve the generalisability of claims regarding the manifestation of the placebo analgesia phenomenon across time.

Given this, one interpretation of our results could be that we have not detected groups of individuals that are consistent placebo responders and non-responders, but rather they are consistent when the context and experimental setup is identical across time (i.e. they might not show similar placebo responses across different paradigms). That is, a consistent placebo responder might have become a consistent non-responder if, for whatever reason, they failed to show placebo on their first trial. In this regard, our results highlight the importance of the initial encounter in a specific context as most important to producing stronger placebo analgesic effects. After this initial encounter, an individual might be ‘locked in’ to a particular response each time they return to that specific context. This holds significant implications for the manifesting of placebo effects in non-experimental settings, essentially that first impressions matter, and raises the possibility that one might be able to shift an individual’s placebo responsiveness based on their first experience.

In conclusion, we provide quantitative evidence for the consistency of placebo analgesic responses and that this consistency is not predicted by psychological measures but rather an individual’s initially recorded placebo response. Placebo responses were associated with altered activation in frontoparietal brain structures that influence the cognitive appraisal of nociceptive information. The results of this study and future work characterising placebo response consistency in humans may motivate modifications to clinical trial run-in phases, with pretesting placebo responsiveness in a design similar to that being employed in the trial, allowing individuals to be categorised in a consistent non-responder group. Limiting the effects of placebo analgesia responses during clinical trials will significantly improve our capacity to determine the effectiveness of novel analgesic medications and the underlying neural circuitry.

## Methods

### Ethics

All experimental procedures were approved by the University of Sydney Human Research Ethics Committee (HREC 2022/590) and were consistent with the Declaration of Helsinki. Written informed consent was obtained from participants prior to each session commencing. Whilst inside the Magnetic Resonance Imaging (MRI) scanner, participants were equipped with an emergency buzzer which could be squeezed to stop the experiment at any time. At the conclusion of all five sessions, participants were informed verbally and via a written statement of the necessary deception and true methodology of the experiment and were invited to seek clarification over any of the procedures they had experienced.

### Participants

Thirty-four healthy control participants were initially recruited via word of mouth and advertisement at an adjacent university campus for the study (18 female, mean age = 21.59 ± 0.44 years [±SEM]; range 19–31 years). Four participants were excluded from final analyses due to three failing to return after the first or second experimental sessions, and the other with dental implants, which irrevocably distorted functional brain imaging data. An a priori power analysis using results from a previous behavioural study investigating behavioural consistency of placebo analgesia revealed a sample size of 21 would be necessary to detect similar effects with 90% power (*d* = 0.67, *α* = 0.05, power = 0.9)^15,58^. Furthermore, power analysis using NeuroPower^59^ indicated that our total sample size of 30 participants provides 83% power under Bonferroni correction (*α* = 0.05).

### Experimental design

The study involved five separate experimental sessions being conducted as close to 14 days apart as possible. Participants were scheduled as close to the same day and time as possible for return visits to ensure the environment and participants’ routine when conducting the study were kept as similar as possible. The study was disguised to participants as investigating the effectiveness of a cream labelled 'XK50.113'—described as a novel analgesic agent (in reality, a placebo)—capable of specifically influencing thermal pain in consistently providing pain relief. Participants were informed that XK50.113 had been tested in animals, but its effect on humans was yet unknown, and as such, the experiment required them to attend repeatedly with the session protocol kept as similar as possible between visits. Each session lasted roughly 90 min and consisted of a conditioning and test phase—with conditioning conducted in a clinical side room adjacent to the MRI scanner, and test conducted with participants inside the MRI scanner itself. Throughout the study, noxious thermal stimuli were administered to the volar surface of participants’ left and right forearms using an MR-compatible Peltier-element thermode (4.5 cm^2^ stimulation surface), which delivered stimuli at pre-programmed temperatures via a thermal cutaneous stimulator (TCS II, QST-labs). Each stimulus lasted 12 s, including a short (<1 s) ramp-up period (fifty degrees per second), a plateau period, and a short (<1 s) ramp-down period (fifty degrees per second). Each stimulus was separated by a 12 s inter-trial interval at a non-painful baseline of 32 degrees Celsius. Throughout conditioning, participants rated their pain continuously using a horizontal visual analogue scale (VAS), controlled with their index finger, ranging between 0 and 100 (0 = no pain at all, 100 = worst tolerable pain).

Whilst inside the MRI scanner, participants continuously reported their perceived pain by operating a button box in their left hand, which controlled the position of a slider mimicking the VAS used outside the scanner, reflected on a digital screen overhead and visible to participants at the end of the magnet bore. In the 24 h preceding their first experimental session, participants were sent a secure link to a series of questionnaires hosted on University Redcap servers and asked to complete the life orientation test-revised form (LOT-R), the behavioural inhibition and avoidance scales (BIS/BAS), the trait version of the state-trait anxiety inventory (STAI-T), the pain catastrophizing scale (PCS), and the short form of the EPQ. Immediately prior to the conditioning phase of session 1, participants completed the state version of the state-trait anxiety inventory (STAI-S).

### Conditioning

Conditioning began by the researcher applying both the XK50.113 placebo as well as a control cream (labelled and described to be an inert moisturiser) of similar colour and consistency to XK50.113 to adjacent regions of the right forearm. The experimenter wore gloves and used applicators to apply the creams to enhance the believability that XK50.113 contained active ingredients. The locations of these two creams were counterbalanced between both participant and session (that is, each participant would have either three sessions with the placebo placed proximal to the control and two sessions with the control placed proximal to the placebo or vice versa) to reduce any confounders of skin site or distal versus proximal sensitivity effects influencing pain rating response profiles. These creams were left to 'soak in and take effect' for 5 min during which thermal calibration was conducted. This test consisted of the application of 14 noxious stimuli in randomised order, being applied to the volar left forearm, ranging between 44 and 48.5 degrees Celsius in 0.5-degree intervals. Participants were instructed to provide a verbal rating out of 10 during each stimulus presentation, which the experimenter would use to determine the temperature that elicited moderate pain and be used on both creams throughout the remainder of the session. In reality, we recorded two temperatures—one eliciting a low pain response (2–3/10) and one eliciting a moderate pain response (4–5/10). With these temperatures recorded, a standard response conditioning paradigm was then conducted, whereby lower temperature stimuli were deceptively applied to the XK50.113 placebo cream relative to the control moisturiser. Each cream site received two series of 10 noxious stimuli sequentially, with a 5-min break between series to eliminate lingering sensitivity, with participants rating their pain continuously using the VAS. Participants were informed that both cream sites were receiving identical intensity stimuli throughout this period, initiating the belief that any difference in perceived pain was due to XK50.113 taking effect (Fig. 1B).

### Test phase

Once both cream sites had received two series of stimuli, we re-applied the creams and guided participants to the MRI suite. During the test session, we collected a T1-weighted anatomical image and two functional MRI (fMRI) sequences where the stimulator was attached to first the control and then the XK50.113 placebo cream in succession. Dissimilar to conditioning, during the test phase both the control and placebo creams received an identical stimulus intensity at the moderate temperature elected by participants, such that any reduction in pain during this phase on the XK50.113 relative to the control site indicated a placebo analgesia response (Fig. 1B). Once an fMRI sequence had been collected during noxious stimulation of both the control and XK50.113 placebo cream sites, participants exited the scanner, provided verbal confirmation to the researcher for the time and date of their next session, and received a $50 gift card as compensation for their time and travel to complete the session. Once a participant had completed all five sessions, a one-on-one meeting was scheduled between them and the experimenter to debrief and provide an opportunity to ask any questions or raise comments surrounding the study methodology and purpose.

### Behavioural data processing and group assignment

Each participant’s test phase pain rating data were entered into a permutation procedure to determine significant differences in pain ratings provided during stimulation of the XK50.113 placebo cream relative to the adjacent control cream (bootstrapped to 10,000 simulated samples with replacement). Specifically, we determined if the bootstrapped mean pain perceived during placebo cream stimulation was significantly lower than the pain expressed during control cream stimulation. Based on these data, participants were assigned as either a placebo responder or a non-responder to each of the five sessions. If across all five sessions, participants expressed significant placebo analgesia in four or more sessions, they were considered a consistent placebo analgesia responder. If they responded one or fewer times, they were considered a consistent non-responder. Participants that responded either two or three times from the five sessions were considered variable placebo analgesia responders (Fig. 1C, D). Paired t-tests were used to determine the magnitude of placebo analgesia expressed within each session of each group, as well as single-factor ANOVAs to determine differences in temperatures applied throughout conditioning and test phases to evaluate any relation to the expression of placebo analgesia and baseline thermal sensitivity.

### Questionnaire data interpretation and model generation

Coded questionnaires were scored using their respective scoring sheets, and responses tallied into a data table entered into Graphpad Prism version 10.1.2 for Windows (GraphPad Software, Boston, Massachusetts USA). Principal component analysis was conducted with the variable of interest entered as the number of times out of the five sessions participants elicited a significant placebo response. Questionnaire data with a loading score to the first principal component greater than 0.7 was retained and entered into the Matlab Classification Learner toolbox (Fig. 2A). This reduced feature set was trained using all Quick-To-Train model types and performance compared. Confusion matrices and Receiver Operating Characteristic Curves were generated for the optimal model to visualise the ability of psychological information in predicting the consistency of placebo analgesia responses.

### MRI data acquisition and preprocessing

Brain images were acquired at 3-Tesla using a 32-channel digital head coil (Ingenia CX, Philips, Best, The Netherlands). Participants were positioned supine with their head in the coil and sponges placed either side to support the head laterally and minimise motion. A T1-weighted anatomical image set covering the whole brain was collected as part of the first experimental session (repetition time = 7.2 ms, echo time = 3.4 ms, raw voxel size = 1.0 × 1.0 × 1.0 mm, 170 sagittal slices, scan time = 5 min). During each experimental visit, two functional MRI sequences were also collected—one with the thermode positioned over the control cream site and the other with the thermode positioned over the XK50.113 placebo cream site. Each sequence consisted of 6 dummy and 246 dynamic gradient echo echo-planar measurements using BOLD contrast covering the entire brain. Images were acquired in an interleaved collection pattern with a multiband factor of 3 (repetition time = 1500 ms, echo time = 30 ms, raw voxel size = 2.0 × 2.0 × 2.0 mm, 75 axial slices, scan time = 6 min 18 s).

Image preprocessing and statistical analyses were performed using the statistical parametric mapping toolbox (SPM12)^60^ in MATLAB Version 2023a. The first five dynamic volumes were removed due to signal saturation, resulting in 241 functional volumes being processed. These images were slice-timing and motion-corrected, with the parameters visually inspected for volume-to-volume motion exceeding 1 mm in rotational or 0.5 degrees in translational planes. No participant displayed exaggerated motion in any of the five sessions, and as such, all image series remained included for further analyses. Images were then linearly detrended to remove global signal drift, passed through the DRIFTER toolbox^61^ to remove physiological noise relating to cardiac (60–120 beats per minute +1 harmonic) and respiratory (8–25 breaths per minute +1 harmonic) frequency, and noise relating to volume-to-volume movement was removed by attaching the motion parameter files as regressors following a linear modelling of realignment parameters procedure^62^. Each participant's fMRI image series from the five sessions were then resliced into 1.0 mm isotropic voxels and co-registered to the corresponding T1-anatomical image collected in the first session. The T1 image was then normalised to the Montreal Neurological Institute (MNI) template, and these parameters applied to each of the functional image series. Normalised functional image series were finally spatially smoothed using a 6 mm full-width at half maximum Gaussian filter.

### fMRI statistical analysis

A repeating boxcar model convolved with a canonical hemodynamic response function was applied to each participant's fMRI series across the five time points. Within this model, volumes where noxious stimuli were applied were assigned a value of '1' and inter-trial-interval volumes '0'. The resulting 1st-level contrast images were then used in group-level analyses. A main effect of pain analysis was first conducted through the construction of 1 × 5 full factorial design using the control cream site contrast image in all 30 participants. Next, a 30 × 2 × 5 flexible factorial design was constructed, with 'participant' (thirty layers), 'cream site' (two layers) and 'time' (five layers) entered as separate factors. For this design, contrast images encoding neural activation during thermal stimulation of either the control or XK50.113 placebo creams across the five sessions were entered into respective cells with a response covariate included per session ('0' = no response, '1' = placebo response). Brain regions demonstrating a significant main effect of placebo in all participants were saved as a volume-of-interest (VOI) mask and used in subsequent analyses to investigate brain changes that occurred in consistent placebo analgesia responders. That is, to determine the associated changes in neural activity that related specifically to placebo analgesia response consistency, a 2 × 5 full factorial design was constructed in SPM12 with both cream and time entered as factors, and the overall main effect of placebo thresholded VOI image included as an inclusion mask. Main effect and interaction contrasts were generated, and the main effect of cream F-contrast split into positive and negative t-contrasts to visualise brain regions showing consistent increases and decreases in neural activity across time relating to placebo response expression, respectively. An interaction contrast of consistent responders versus non-responders was also generated to investigate circuitry working in opposing directions between response groups. These contrast maps were visualised at an uncorrected threshold of *p* < 0.001 with a cluster extent threshold applied to the flexible factorial analysis of 50 contiguous voxels. Small volume correction was applied to each resulting cluster using corresponding parcels from the extended human connectome project atlas (HCPex) to reduce the likelihood of type II errors^63^.

Each cluster surviving small-volume correction in both the whole cohort or consistent placebo responder main effect of placebo analyses were then saved as a VOI mask, and beta-values extracted from each fMRI series at each time point in each of the consistent placebo responder, non-responder and variable responder groups. These values were entered into post hoc repeated ANOVA’s within GraphPad with Geisser-Greenhouse correction applied to visualise the magnitude and directionality of signal change at each time point. Specifically, in the consistent placebo responder analysis, for each resulting cluster, 2 × 2 × 5 three-way repeated measures ANOVA was also performed using the values of consistent responders, variable responders and non-responders to determine the presence of group × cream interactions over time.

### Statistics and reproducibility

First, bootstrapped permutation was performed on test phase behavioural data during noxious stimulation of XK50.113 placebo or control cream sites (*n* = 30), with significant differences defined at a corrected threshold of *p* < 0.05. Cohen’s D effect size estimates and paired t-tests were conducted at a main effect and on each participant group, respectively, to quantify differing magnitudes of placebo analgesia generated within- and between each of the five experimental sessions. Single-factor ANOVA was used to investigate whether group assignment as a consistent responder, variable responder, or consistent non-responder was associated with differences in thermal sensitivity, age, or sex assigned at birth. Each psychological questionnaire was scored using it’s own scoring sheet, and both single-factor ANOVA and linear regression conducted using these questionnaire values to identify group-level or linear associations between single psychological measurements and placebo analgesia responsivity (*p* < 0.05). Principal component analysis was performed to identify the combination of questionnaires contributing most strongly to greater placebo analgesia response frequency, with the MATLAB classification learner toolbox then utilised to fit a predictive model using these questionnaire scores. Three separate functional brain imaging analyses were conducted, each at a statistical threshold of *p* < 0.001, with a cluster-forming threshold of 50 contiguous voxels. First, a main effect of pain analysis was conducted using each individual’s contrast image encoding noxious input to the control cream site (*n* = 30) over time (5 sessions). Second, a main effect of placebo analysis was conducted as a flexible factorial analysis between each individual’s contrast image encoding noxious input to the XK50.113 relative to the control cream site (*n* = 30) over time (5 sessions). From the second analysis, thresholded regional brain activity maps were saved as VOI masks and applied in a full factorial analysis using only consistent placebo responders (*n* = 14), to determine where the main effect of placebo was dependent on selectively mounting a significant analgesic response over time (5 sessions). Resulting clusters were saved, with beta-values extracted from the alternate two-groups contrast images, and a post-hoc three-way ANOVA conducted between groups and over time to determine significant differences in brain activation related to placebo analgesia responses between consistent responders (*n* = 14), variable responders (*n* = 7) and consistent non-responders (*n* = 9).

### Reporting summary

Further information on research design is available in the Nature Portfolio Reporting Summary linked to this article.

## Supplementary information

Transparent Peer Review File

Supplementary Materials

Description of Additional Supplementary Files

Supplementary Data

Reporting Summary

## Data Availability

Raw and coded behavioural, psychological and both main effect and responder-specific effect functional imaging cluster data supporting these findings can be found in the associated Supplementary Data Excel document. Further data are available from the corresponding author (LAH), upon reasonable request.

## Code availability

All code required to generate the findings of this study are available through public download of MATLAB Version 2023a and listed toolboxes found within the 'Method' section, Statistical Parametric Mapping Version 12, MRIcroGL and Graphpad Prism version 10.1.2.
