# Supplementary material for: Placebo analgesia persistence: evaluating behavioural stability in repeated trials
Source: Commun Biol. 2026 May 28;9:1133. doi: 10.1038/s42003-026-10377-8 (PMC13500666; doi:10.1038/s42003-026-10377-8)
Supplement: Supplementary file 1 [file 42003_2026_10377_MOESM1_ESM.pdf]

nature portfolio

Peer Review File

Placebo Analgesia Persistence: Evaluating Behavioural Stability in Repeated Trials

Corresponding Author: Professor Luke Henderson

This file contains all reviewer reports in order by version, followed by all author rebuttals in order by version.

Version 1:

Reviewer comments:

Reviewer #1

(Remarks to the Author)
I previously reviewed this paper in another journal and had no further suggestions. The authors have addressed the suggestions of the additional reviewer.

Reviewer #2

(Remarks to the Author)
This revised manuscript incorporates previous reviewers' comments and, as such, has improved. Still, an important issue raised in the past needs to be addressed more effectively. The authors continue to assume that the specific design used here, acute thermal stimulus responses to placebo conditioning, is sufficient to be used in the clinical setting and that their study addresses clinical studies. They ignore a series of studies that have been performed in the clinical setting of patients with chronic pain. These studies address predictability of placebo response when psychological and brain properties are combined, see around 6 studies by Apkarian group. The present study needs to be properly contrasted with the latter as there are substantial differences between them.

Open Access This Peer Review File is licensed under a Creative Commons Attribution 4.0 International License, which permits use, sharing, adaptation, distribution and reproduction in any medium or format, as long as you give appropriate credit to the original author(s) and the source, provide a link to the Creative Commons license, and indicate if changes were made.
In cases where reviewers are anonymous, credit should be given to 'Anonymous Referee' and the source.
The images or other third party material in this Peer Review File are included in the article's Creative Commons license, unless indicated otherwise in a credit line to the material. If material is not included in the article's Creative Commons license and your intended use is not permitted by statutory regulation or exceeds the permitted use, you will need to obtain permission directly from the copyright holder.
To view a copy of this license, visit https://creativecommons.org/licenses/by/4.0/

Dear editor,

We thank the editor for the opportunity to modify the manuscript in response to reviewers comments.

Please find below response to reviewer 3 conceptual concerns which was provided previously during resubmission on 10th of March 2026. We have additionally already provided both marked and clean copies of the manuscript with the changes outlined responding to this conceptual concern during the previous resubmission. We hope that our changes address any concerns. Attached now are a marked up and clean copy of the manuscript, with the below changes made to the previous conceptual concern of reviewer 3 still present as mark ups in the "marked up" version, alongside the additional changes now made to figures 3 and 4, and the data availability statement.

We thank the reviewer for providing their overall interpretation of the manuscript as adapted through comments provided by reviewers #1 and #2, which have now been implemented and resolved to both reviewers' satisfaction.

Overall, we interpret the text provided by reviewer #3 as three overall suggested changes: 1) reduce magnitude of claims made regarding clinical utility of the findings; 2) alter introductory section to acknowledge prior evidence of brain imaging investigations utilizing sham topical ointments and response conditioning to evoke placebo analgesic effects in a single session; 3) amend limitations section to acknowledge that these findings are constrained to when the conditioning model and placebo substance are kept identical across sessions, and may not necessarily translate to consistency when these methodological factors are altered between sessions.

Before addressing each of the above, we would make it clear that the statement "Overall the novel information we garner from this study remains minimal" is categorically not accurate and without basis. Broadly, this work presents the first investigation to explore placebo analgesia response consistency, and the first of its kind to assess response consistency and measure brain activity concurrently. We are aware of only two similar works - (Lasagna,

Mosteller, von Felsinger, & Beecher, 1954) and (Whalley, Hyland, & Kirsch, 2008). The former was conducted over 70 years ago, in a clinical setting, with no brain imaging or with the updated knowledge of the central tenets of mounting the placebo analgesia phenomenon in humans. The latter, while more recent, utilized only two sessions, without brain imaging, and made the case for which the reviewer uses as evidence for responders not necessarily mounting a response when the sham substance is altered in appearance or description. This work does not explore what happens when variables of this kind are altered as it was critical to us to first establish whether placebo behaviours were consistent at all across a time and session length which we provide here. We do however agree that future work leveraging the paradigm we establish here where certain experimental factors are altered between sessions would be of relevance, and changes have been made to the manuscript to acknowledge this as an extension of the novel work we present in this manuscript.

The following changes have been made to the manuscript in line with the three points above which again are our interpretation of the comment provided, we would be happy to further exchange ideas with the reviewer as to additional changes which can be made to align the manuscript introduction and discussion with the results presented, to ensure there is a match between conclusions drawn as results presented.

1) reduce magnitude of claims made regarding clinical utility of the findings

We had originally intended for these findings to be more relevant for clinical trial research settings, where a placebo control group is always found – and as such agree the use of the word “clinical setting” can potentially be misconstrued and over-interpretive of the data. We have now amended this word choice to “non-experimental settings” since most of the statements where clinical settings were used were general in nature, this phrasing allows interpretation to the general tenets of placebo effects rather than claiming their association within clinics. The following changes have been made to reduce the magnitude of claims made relative to the level of support provided by these data, especially in a clinical setting.

Lines 17-18: “and by extension, ~~utility~~ consideration in non-experimental ~~clinical~~ settings.”

Lines 388-392: "In this regard, our results highlight the importance of the initial encounter in a specific context ~~(be it an experimental paradigm like ours, a clinical visit or clinical trial)~~ as ~~the~~ most important to producing stronger placebo analgesic effects. After this initial encounter, an individual might be 'locked in' to a particular response each time they return to that specific context. This holds significant implications for the manifesting of placebo effects in non-experimental settings ~~clinical contexts~~,"

Lines 398-402: "The results of this study and future work characterising placebo response consistency in humans may motivate modification to clinical trial run-in phases ~~can potentially change the way in which we conduct clinical trials~~, with pretesting placebo responsiveness in a design similar to that being employed in the trial …"

2) *alter introductory section to acknowledge prior evidence of brain imaging investigations utilizing sham topical ointments and response conditioning to evoke placebo analgesic effects in a single session*

We have amended the introduction and hypotheses section of the manuscript to make specific statements relevant to single session inductions of placebo analgesia in response to the topical response conditioning design. The general nature of our original hypotheses was motivated by the fact that there is no available literature on consistency patterns of brain activation across time in the context of placebo analgesia.

Lines 65-71: "Of these cortical nodes, the dlPFC has received particular attention in recent years, commonly activated through response conditioning of placebo analgesia using sham substances or through social observation (Eippert et al., 2009; Freeman et al., 2015; Schenk & Colloca, 2020; Wager et al., 2004), and flexibly altering placebo analgesia expression when subjected to non-invasive brain stimulation (Krummenacher, Candia, Folkers, Schedlowski, & Schönbächler, 2010; Tu et al., 2021). Moreover, directed connectivity analyses leveraging functional brain imaging data have shown both direct projections from the dlPFC and via relay with the anterior cingulate as the predominant cortical-brainstem pathway associated with stronger placebo analgesia responses (Crawford et al., 2023; Sevel, Craggs, Price, Staud, & Robinson, 2015). Importantly, ~~this same~~ the brainstem circuitry recruited by these cortical

circuits is thought to be involved in mediating the effects of most analgesic drugs including opiates (Chieng & Christie, 1994) and cannabinoids (Manzanares, Julian, & Carrascosa, 2006)."
Lines 90-92: "driven by changes in neural activity consistent with brain regions associated with single-trial expression of placebo analgesia such as the dlPFC and anterior cingulate cortices ~~in pain modulatory brain regions and associated cognitive areas~~."

3) *amend limitations section to acknowledge that these findings are constrained to when the conditioning model and placebo substance are kept identical across sessions, and may not necessarily translate to consistency when these methodological factors are altered between sessions*

We have now expanded on the section of the limitations section where we address the fact that the present findings can only explain expression of placebo analgesia longitudinally when experimental factors such as conditioning stimulus and noxious input type are also kept consistent over time.

Lines 389-399: "Importantly, a feature of our experimental design is that each participant was assessed in the same environment (inside an MRI scanner) and using the same placebo paradigms during each experimental session. As such, our findings are constrained to only associating these consistency behaviours of placebo analgesia within a response conditioning model using a deceptively described sham topical ointment and using short-lasting thermal noxious stimuli. Moreover, we cannot provide evidence for whether our cohort of consistent responders and non-responders would display these same behavioural patterns if the placebo substance were changed to a pill or in a social observation model, or if the type of stimulus was adjusted to a mechanical or visceral experimental model of pain processing. Future research leveraging the design we have now established, however altering these experimental components during specific session numbers would be of benefit to improve generalizability of claims regarding manifestation of the placebo analgesia phenomenon across time."

References:

Chieng, B., & Christie, M. J. (1994). Hyperpolarization by opioids acting on mu-receptors of a sub-population of rat periaqueductal gray neurones in vitro. *Br J Pharmacol, 113*(1), 121-128. doi:10.1111/j.1476-5381.1994.tb16183.x

Crawford, L. S., Meylakh, N., Macey, P. M., Macefield, V. G., Keay, K. A., & Henderson, L. A. (2023). Stimulus-independent and stimulus-dependent neural networks underpin placebo analgesia responsiveness in humans. *Communications Biology, 6*(1), 569. doi:10.1038/s42003-023-04951-7

Eippert, F., Bingel, U., Schoell, E. D., Yacubian, J., Klinger, R., Lorenz, J., & Büchel, C. (2009). Activation of the Opioidergic Descending Pain Control System Underlies Placebo Analgesia. *Neuron, 63*(4), 533-543. doi:10.1016/j.neuron.2009.07.014

Freeman, S., Yu, R., Egorova, N., Chen, X., Kirsch, I., Claggett, B., . . . Kong, J. (2015). Distinct neural representations of placebo and nocebo effects. *NeuroImage, 112*, 197-207. doi:10.1016/j.neuroimage.2015.03.015

Krummenacher, P., Candia, V., Folkers, G., Schedlowski, M., & Schönbächler, G. (2010). Prefrontal cortex modulates placebo analgesia. *Pain, 148*(3), 368-374. doi:10.1016/j.pain.2009.09.033

Lasagna, L., Mosteller, F., von Felsinger, J. M., & Beecher, H. K. (1954). A study of the placebo response. *The American Journal of Medicine, 16*(6), 770-779. doi:https://doi.org/10.1016/0002-9343(54)90441-6

Manzanares, J., Julian, M., & Carrascosa, A. (2006). Role of the cannabinoid system in pain control and therapeutic implications for the management of acute and chronic pain episodes. *Curr Neuropharmacol, 4*(3), 239-257. doi:10.2174/157015906778019527

Schenk, L. A., & Colloca, L. (2020). The neural processes of acquiring placebo effects through observation. *NeuroImage, 209*, 116510. doi:10.1016/j.neuroimage.2019.116510

Sevel, L. S., Craggs, J. G., Price, D. D., Staud, R., & Robinson, M. E. (2015). Placebo analgesia enhances descending pain-related effective connectivity: a dynamic causal modeling study of endogenous pain modulation. *J Pain, 16*(8), 760-768. doi:10.1016/j.jpain.2015.05.001

Tu, Y., Wilson, G., Camprodon, J., Dougherty, D. D., Vangel, M., Benedetti, F., . . . Kong, J. (2021). Manipulating placebo analgesia and nocebo hyperalgesia by changing brain excitability. *Proc Natl Acad Sci U S A, 118*(19). doi:10.1073/pnas.2101273118

Wager, T. D., Rilling, J. K., Smith, E. E., Sokolik, A., Casey, K. L., Davidson, R. J., . . . Cohen, J. D. (2004). Placebo-induced changes in FMRI in the anticipation and experience of pain. *Science, 303*(5661), 1162-1167. doi:10.1126/science.1093065

Whalley, B., Hyland, M. E., & Kirsch, I. (2008). Consistency of the placebo effect. *J Psychosom Res, 64*(5), 537-541. doi:10.1016/j.jpsychores.2007.11.007

At the same time, we ask that you ensure your manuscript complies with our editorial policies.

Specifically:

For all graphs depicting a single point value (e.g., mean) with error bars, you must add individual data points or convert the graph to a boxplot or dot-plot to show data distribution. Individual data points have now been added to all remaining figures: namely, Figure 3 panel B; and Figure 4 panel B. Adapted figures and figure legends can be found below, as well as in marked and clean versions of the submitted manuscript.

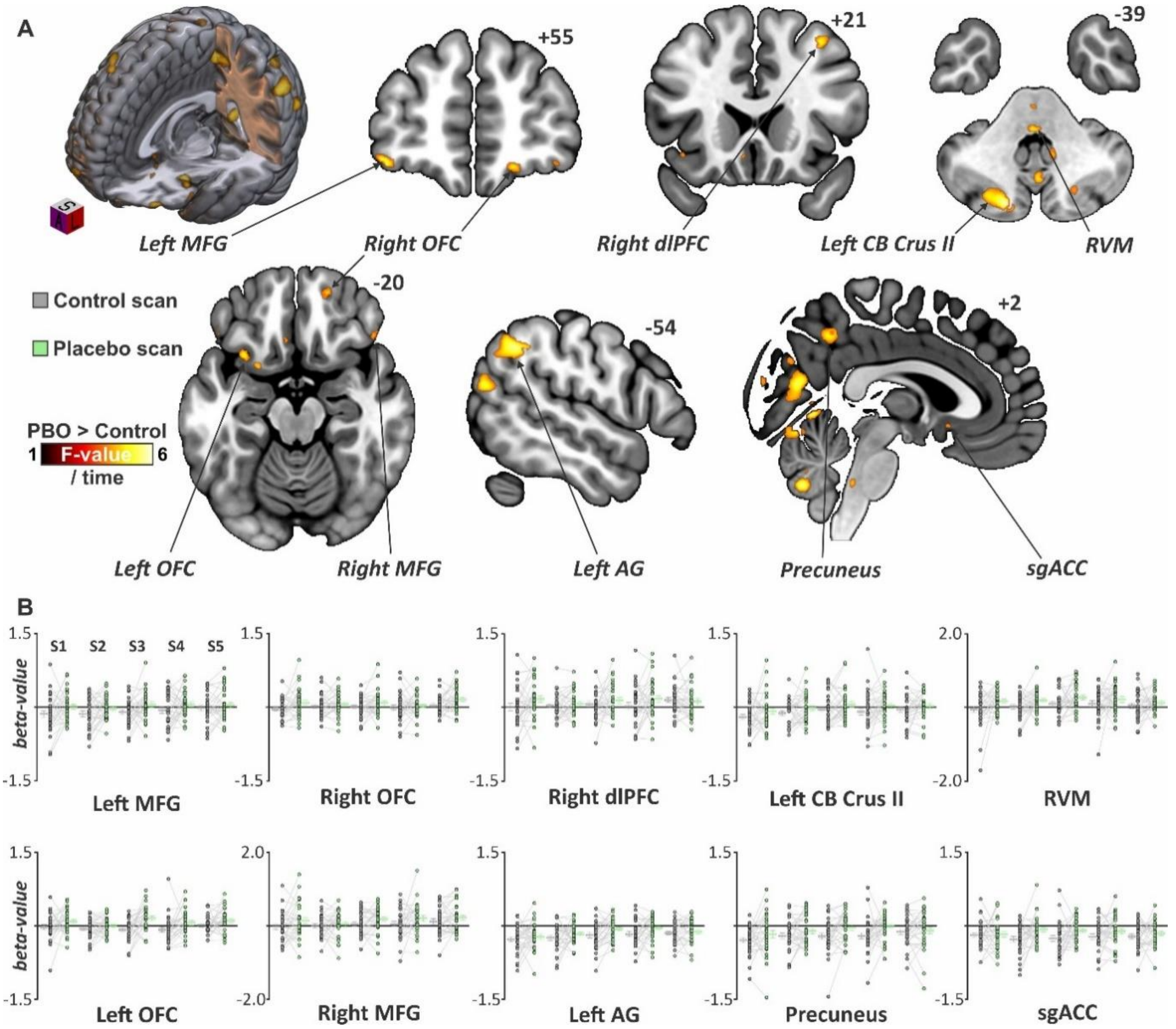

Figure 3. Altered placebo-related brain activity patterns over time. A) Main effect of placebo over time in all participants. A 30x2x5 flexible factorial analysis including factors of "participant", "scan", and "time", was constructed within SPM12 with average perceived pain per scan included as a regressor of interest (n=30). Significant increases in activation were observed within the bilateral middle frontal and orbitofrontal cortices, as well as within the right dorsolateral prefrontal cortex, left angular gyrus and cerebellar crus II, and within the rostroventromedial medulla and subgenual cingulate cortices. **B) Signal intensity change extracted per session as a main effect of placebo.** Extraction of signal intensity values in both the control (gray bars) and placebo (green bars) scans revealed that largely average signal intensity was at baseline during control pain processing, and increased during placebo-related pain encoding in each of the five experimental sessions. Notably, within the left angular gyrus, and both the precuneus and subgenual cingulate cortex, negative signal intensity was observed during control pain processing, which increased but remained negative during placebo-related pain encoding. Average session mean (±SEM) signal intensity during stimulation of the control (gray bar) and XK50.113 placebo (green bar) creams are provided with interconnected dots and lines delineating each participants activity change over time. A navigation cube and rendered image is provided in the upper left corner to aid in visualization of significantly activated regions, with MNI co-ordinates in the upper right corner of each slice in either coronal, axial, or sagittal orientations, respectively. *MFG = middle frontal gyrus; OFC*

= orbitofrontal cortex; CB Crus II = cerebellar crus layer two; RVM = rostral ventromedial medulla; AG = angular gyrus; sgACC = subgenual anterior cingulate cortex.

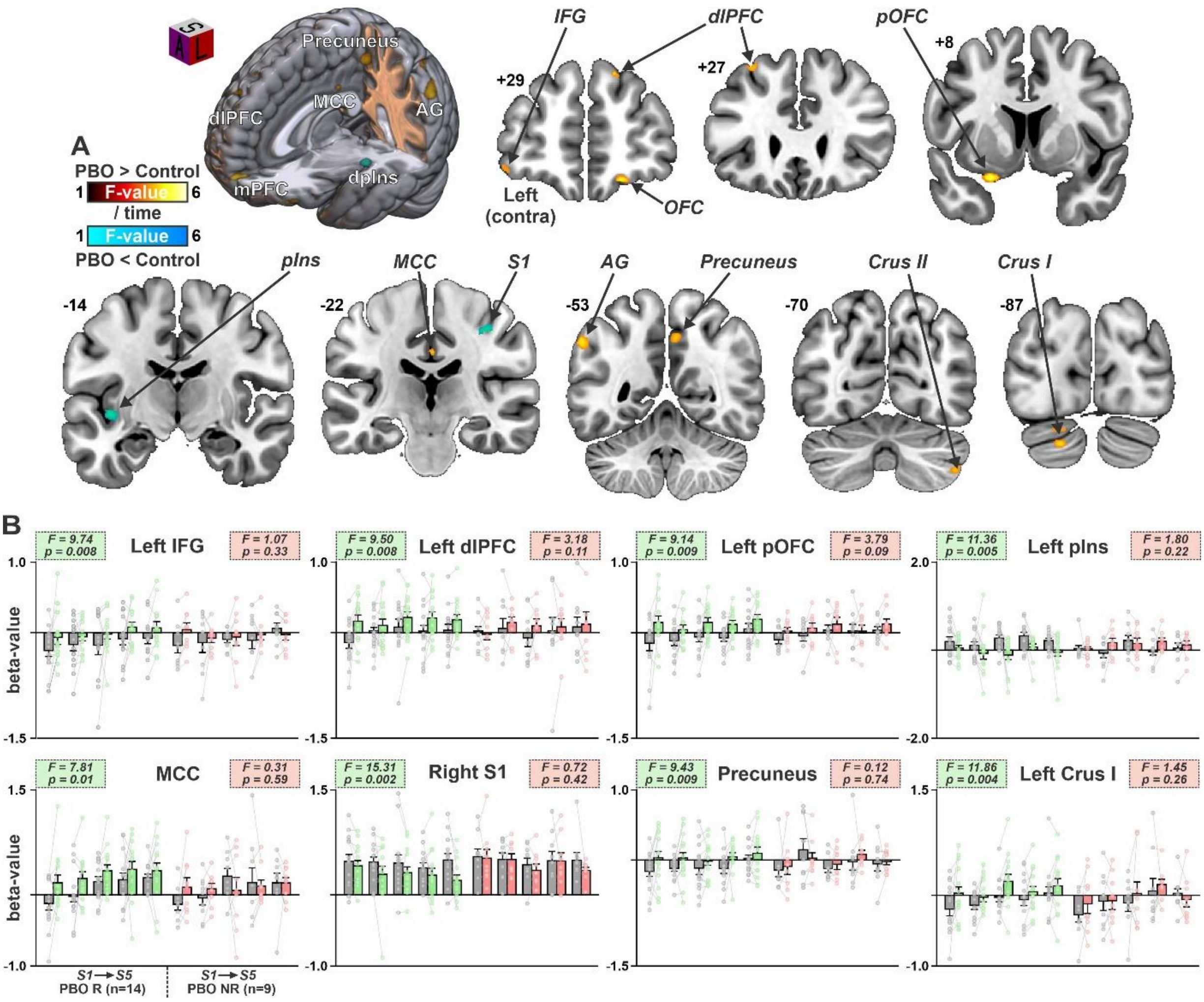

Figure 4. Masked consistent placebo responder main effect of placebo analysis. A) Cortical regions of altered activity during consistent and significant behavioural placebo analgesia. A full factorial analysis conducted on the 14 classified consistent placebo analgesia responders with both "scan" and "time" loaded as separate two and five layer factors, respectively, was conducted using the main effect of placebo contrast map of all participants as a volume of interest mask, thresholding analyses to only cortical regions altered in the context of a placebo administration. This revealed a subset of regions previously identified which significantly increased in activity only in those participants who consistently mounted behavioural analgesia, consisting of the bilateral orbitofrontal and dorsolateral prefrontal cortices, the left inferior frontal and angular gyri, the left cerebellar crus I and the right crus II, and in the midline precuneus and middle cingulate cortex. Additionally, significantly reduced signal intensity during placebo relative to control cream noxious stimulation was identified within the left posterior insula and right primary somatosensory cortex. **B) Signal intensity changes extracted within each consistent responder and non-responder group.** Resulting clusters of consistent responder main effect of placebo changes were saved as volume of interest masks and measures of signal intensity (beta-value) during each functional series extracted from consistent placebo responder and non-responder groups. Post hoc two factor repeated

ANOVA was conducted in each group separately, showing that these significant changes were isolated only to consistent placebo responder groups. Average session mean (±SEM) signal intensity during stimulation of the control (gray bars) and XK50.113 placebo (green and red bars) creams are provided with interconnected dots and lines delineating each participants activity change over time. *IFG = inferior frontal gyrus; dlPFC = dorsolateral prefrontal cortex; pOFC = posterior orbitofrontal cortex; pIns = posterior insula cortex; MCC = middle cingulate cortex; S1 = primary somatosensory cortex; AG = angular gyrus PBO R = consistent placebo responder; PBO NR = consistent placebo non-responder.*

It's mandatory to provide access to the numerical source data for graphs and charts either through a repository or by providing the data in a Supplementary Data file (in excel format).

Source data comprising analyses presented in each graph and chart are now available through the submitted excel document titled "Source data".

The "Data availability" section of the clean and marked-up versions of the manuscript have been adapted as follows:

Lines 605-608: "**Data Availability:** Raw and coded behavioural, psychological, and both main effect and responder-specific effect functional imaging cluster data supporting these findings can be found in the associated source document. Further data are available from the corresponding author (LAH), upon reasonable request"

Dear editor,

We thank the editor for the opportunity to modify the manuscript in response to reviewers' comments.

Please find below response to reviewer 2:

This revised manuscript incorporates previous reviewers' comments and, as such, has improved. Still, an important issue raised in the past needs to be addressed more effectively. The authors continue to assume that the specific design used here, acute thermal stimulus responses to placebo conditioning, is sufficient to be used in the clinical setting and that their study addresses clinical studies. They ignore a series of studies that have been performed in the clinical setting of patients with chronic pain. These studies address predictability of placebo response when psychological and brain properties are combined, see around 6 studies by Apkarian group. The present study needs to be properly contrasted with the latter as there are substantial differences between them.

We thank the reviewer for this comment. We have now clarified this distinction in the manuscript. Whereas Apkarian's work focuses on predicting clinical placebo response from primarily brain-network properties in chronic pain, our study examines the reproducibility and temporal structure of experimentally induced placebo analgesia in a healthy individual, using a paradigm that affords precise control over stimulus intensity, timing, and expectancy induction. To integrate the work of Apkarian into the manuscript we have added the following to the Introduction:

"Consistent with this circuitry, a study comparing two clinical trials in individuals with osteoarthritis found that dlPFC functional connectivity predicts placebo responses [29] and two follow-up studies showed that dlPFC-precentral gyrus functional connectivity predicted placebo responses in two different chronic low back cohorts and osteoarthritis pain patients[30]. In addition to these reported brain activity measures, the ability to classify individuals that display consistent placebo analgesia responses or non-responses, could aid in fundamentally change the way in which we structure clinical trials and greatly improve our capacity to determine novel analgesic medication effectiveness and the underlying neural circuitry."

And the Discussion:

“It is possible that placebo reactions in pain-free individuals differ to those in individuals with chronic pain, however placebo circuitry remains functional in individuals with chronic lower back pain, since chronic pain patients exhibit placebo response rates similar to those of pain-free controls, dlPFC-connectivity strength predicts placebo responsivity during the placebo-arm of clinical trials in individuals with chronic back pain and osteoarthritis chronic pain[6,7,29,30].”

Regards

Luke
