# Supplementary material for: Placebo analgesia persistence: evaluating behavioural stability in repeated trials
Source: Commun Biol. 2026 May 28;9:1133. doi: 10.1038/s42003-026-10377-8 (PMC13500666; doi:10.1038/s42003-026-10377-8)
Supplement: Supplementary file 2 [file 42003_2026_10377_MOESM2_ESM.pdf]

Supplementary Data

Supplementary Table 1. Test phase pain intensity ratings (mean ±SEM VAS) in consistent placebo responders, non-responders, and variable responders.

	**consistent responders**			**consistent non-responders**			**variable responders**		
	**control**	**"XK50.113" placebo**	**p-value**	**control**	**"XK50.113" placebo**	**p-value**	**control**	**"XK50.113" placebo**	**p-value**
session 1	52.44±3.38	41.61±2.64	<0.001	43.75±4.07	49.58±5.20	0.12	59.60±2.37	56.16±5.15	0.54
session 2	53.63±3.03	35.93±2.86	<0.001	51.01±3.59	50.35±4.33	0.72	60.53±5.31	47.21±4.91	0.005
session 3	49.70±3.67	29.60±3.29	<0.001	52.21±4.68	48.73±4.07	0.45	57.12±3.19	52.86±6.12	0.50
session 4	50.16±2.36	34.30±2.89	<0.001	51.49±1.94	48.92±2.81	0.48	59.16±2.30	45.82±5.56	0.03
session 5	47.78±3.56	31.41±3.19	<0.001	44.67±3.93	49.85±2.83	0.28	47.98±2.16	44.22±3.84	0.19
mean	50.74±1.47	34.57±1.42		48.63±1.77	49.49±1.77		56.88±1.66	49.26±2.44	

Supplementary Table 2. Calibrated low and moderate temperatures in consistent placebo responders, non-responders, and variable responders.

	**consistent responders**		**consistent non-responders**		**variable responders**		**ANOVA**	
	low	moderate	low	Moderate	low	Moderate	low	moderate
session 1	46.32±0.17	47.50±0.14	45.56±0.31	46.89±0.31	46.36±0.22	47.21±0.17	F(2,27)=3.35, P=0.06	F(2,27)=2.09, p=0.14
session 2	46.50±0.13	47.54±0.13	46.17±0.32	47.11±0.30	46.29±0.17	47.21±0.17	F(2,27)=0.65, P=0.53	F(2,27)=1.25, P=0.30
session 3	46.46±0.16	47.43±0.14	45.94±0.37	47.00±0.27	46.07±0.07	47.00±0.10	F(2,27)=1.39, P=0.26	F(2,27)=1.78, P=0.19
session 4	46.32±0.16	47.39±0.15	46.00±0.22	47.06±0.21	46.07±0.12	47.00±0.10	F(2,27)=0.87, P=0.43	F(2,27)=0.24, P=0.24
session 5	46.42±0.15	47.36±0.15	45.67±0.35	46.94±0.20	45.86±0.26	46.79±0.14	F(2,27)=1.87, P=0.17	F(2,27)=2.96, P=0.07
mean	46.44±0.07	47.44±0.06	45.87±0.15	47.00±0.12	46.13±0.09	47.04±0.07		

Supplementary Table 3. Elected low and moderate temperature selected by each participant. *CR = consistent responder, VR = variable responder, CNR = consistent non-responder.*

			**Low Temp (°C)**					**Moderate Temp (°C)**				
**Participant**	**Sex at birth**	**Group Assigned**	**S1**	**S2**	**S3**	**S4**	**S5**	**S1**	**S2**	**S3**	**S4**	**S5**
**1**	M	CR	45.5	45.5	46.5	46.5	46	47	47	47.5	47.5	47
**2**	M	CR	47	46.5	47	47	47	48.5	48	48	48.5	48
**3**	F	CR	45.5	45.5	45.5	46	46	46.5	46.5	46.5	47	47.5
**4**	M	CR	46	47	46.5	46.5	47	48	48	47.5	47.5	48
**5**	F	CR	47	47	47.5	46	46.5	48	48	48	47	47.5
**6**	M	CR	46.5	46.5	46.5	45.5	46.5	47.5	47.5	47.5	47	47.5
**7**	M	CR	46.5	46.5	46	46.5	46.5	47.5	47	47	47.5	47.5
**8**	F	CR	45.5	46	46.5	45	45.5	47	47	47.5	46.5	46.5
**9**	F	CR	45.5	47	47	46	46	47	48	48	47	47.5
**10**	F	CR	46.5	46.5	46	46.5	47	47.5	47.5	47	47.5	48
**11**	F	CR	47.5	47	47	47.5	47.5	48.5	48	48	48.5	48.5
**12**	M	CR	46	46.5	46	46.5	46.5	47	47.5	47	47.5	47.5
**13**	F	CR	46.5	46.5	45.5	46	46.5	47.5	47.5	46.5	47	47.5
**14**	F	CR	47	47	47	47	47.5	48	48	48	48	48
**15**	M	CNR	45	46.5	46.5	46.5	46	47.5	48	47.5	47.5	47
**16**	M	CNR	45	45.5	46.5	46.5	46.5	46.5	47	47.5	47.5	47.5
**17**	M	CNR	45	46	47	45.5	45	46.5	47	47.5	46.5	46.5
**18**	F	CNR	46	46	46.5	46.5	46.5	47	47	47	47.5	47.5
**19**	M	CNR	46.5	47.5	45.5	46.5	45.5	48	48	47	47	47
**20**	F	CNR	46.5	47	46	46.5	46	47.5	48	47	47.5	47
**21**	F	CNR	47	47	46.5	45.5	46	48	48	48	47.5	47.5
**22**	M	CNR	44	44	43	44.5	43	45	45	45	45.5	45.5
**23**	F	CNR	45	46	46	46.5	46.5	46	47	47	47.5	47.5
**24**	F	VR	46	46	46	46	46	47	47	47	47	47.5
**25**	F	VR	45.5	45.5	46	45.5	45	46.5	46.5	46.5	46.5	46.5
**26**	F	VR	46.5	47	46	46.5	47	47.5	48	47	47	47.5
**27**	F	VR	47.5	46.5	46	46	46	48	47.5	47	47	47
**28**	M	VR	46	46.5	46.5	46	45	47	47.5	47.5	47	46.5
**29**	F	VR	46.5	46.5	46	46.5	46.5	47	47	47	47	47
**30**	F	VR	46.5	46	46	46	45.5	47.5	47	47	47	47

Supplementary Table 4. Between-group age and sex differences in consistent placebo responders, consistent non-responders, and variable responders.

	**Consistent Responders (n=14)**	**Consistent Non-responders (n=9)**	**Variable Responders (n=7)**	**F-stat**	**p-value**
**Age (±SEM)**	22.85±0.80	20.67±0.41	21.43±0.34	2.60	0.09
**Male : Female ratio**	6:8	5:4	1:6	1.44	0.26
**Average number of responses**	4.57	0.44	2.43	173.77	<0.001

Supplementary Table 5. Questionnaire data and significance between consistent placebo responders, non-responders, and variable responders.

	**consistent responders**	**consistent non-responders**	**variable responders**	**ANOVA**
LOT-R	16.29±1.19	14.22±1.86	13.71±1.64	F(2,27)=0.78, p=0.46
BIS	20.57±1.12	22.22±0.97	24.43±1.05	F(2,27)=2.52, p=0.10
BAS	40.36±1.53	39.33±1.42	40.00±1.09	F(2,27)=0.11, p=0.89
STAI-S	41.29±3.04	43.33±1.42	46.00±2.82	F(2,27)=0.46, p=0.64
STAI-T	25.07±2.81	28.33±3.06	32.71±3.18	F(2,27)=1.33, p=0.28
PCS	15.14±2.82	14.56±3.77	12.71±2.72	F(2,27)=0.12, p=0.89
E_Psychoticism	2.71±0.41	2.22±0.54	1.86±0.43	F(2,27)=0.76, p=0.47
E_Extraversion	6.79±1.18	8.88±0.88	7.86±1.30	F(2,27)=0.79, p=0.46
E_Neuroticism	5.00±1.14	6.00±1.17	8.00±1.09	F(2,27)=1.34, p=0.28
E_Lie	3.71±0.51	3.33±0.67	4.29±0.77	F(2,27)=0.42, p=0.66

Supplementary Table 6. Psychological questionnaire linear relationships with placebo responsivity overall mean and per session. *LOT-R = life orientation test – revised; BIS = behavioural inhibition scale; BAS = behavioural activation scale; STAI-S = state and trait anxiety index – state; STAI-T = state and trait anxiety index – trait; PCS = pain catastrophizing scale; E_Neur = neuroticism subscale of the Eysenck personality inventory; E_Psyc = psychoticism subscale of the Eysenck personality inventory; E_Ext = extraversion subscale of the Eysenck personality inventory; E_Lie = lie subscale of the Eysenck personality inventory .*

	**Session 1 R-value**	**Session 2 R-value**	**Session 3 R-value**	**Session 4 R-value**	**Session 5 R-value**	**Overall R-value**
**LOT-R**	0.26	-0.02	-0.03	0.15	0.07	0.12
**BIS**	-0.32	0.08	-0.03	-0.20	-0.03	-0.14
**BAS**	0.22	0.09	-0.03	0.08	-0.01	0.09
**STAI-S**	-0.36	0.22	0.14	0.08	0.06	0.06
**STAI-T**	**-0.37***	-0.02	-0.02	-0.22	-0.01	-0.18
**PCS**	-0.17	-0.11	0.05	0.14	0.19	0.03
**E_Neur**	-0.32	0.06	0.00	-0.12	0.11	-0.06
**E_Psyc**	0.20	0.10	0.07	0.03	0.23	0.19
**E_Ext**	0.04	-0.14	-0.28	-0.06	-0.12	-0.19
**E_Lie**	-0.16	0.08	-0.04	-0.24	0.16	-0.05

Supplementary Table 7. Location, level of significance, and cluster size of regions where signal intensity change significantly differed in all participants between control and placebo scans over time as a function of perceived pain. Co-ordinates are in Montreal Neurological Institute (MNI) space.

	MNI co-ordinates [x y z]			Cluster size	Z-score	Control beta value [±SEM]	Placebo beta value [±SEM]	ANOVA all participants
Placebo main effect								
rostral ventromedial medulla	-1	-39	-39	121	3.61	0.04±0.03	0.19±0.03	F[1,29] = 8.25, p=0.007
Cerebellar vermis	2	-67	-41	941	4.15	0.07±0.02	0.20±0.02	F[1,29] = 20.59, p<0.001
Left cerebellar crus II	-22	-79	-37	2345	4.47	-0.08±0.02	0.07±0.03	F[1,29] = 25.89, p<0.001
Right cerebellar crus II	14	-80	-32	297	3.21	-0.09±0.03	0.01±0.02	F[1,29] = 15.53, p<0.001
Left posterior orbitofrontal cortex	-22	8	-20	605	4.70	-0.04±0.02	0.09±0.02	F[1,29] = 15.25, p<0.001
Right inferior frontal gyrus	48	27	-16	89	3.47	-0.13±0.02	-0.02±0.03	F[1,29] = 11.91, p=0.001
Subgenual cingulate cortex	-6	19	-11	81	3.16	-0.23±0.02	-0.12±0.02	F[1,29] = 10.19, p=0.003
	0	15	-8	120	3.38	-0.06±0.02	0.02±0.02	F[1,29] = 10.09, p=0.003
Right orbitofrontal cortex	17	53	-16	753	3.78	0.01±0.02	0.10±0.02	F[1,29] = 7.75, p=0.009
Right middle frontal gyrus	37	56	-14	56	3.26	0.04±0.03	0.16±0.03	F[1,29] = 7.88, p=0.009
Left middle frontal gyrus	-41	54	-14	174	4.08	-0.09±0.03	0.03±0.02	F[1,29] = 13.79, p<0.001
Calcarine + Precuneus	-13	-78	-10	8645	4.47	-0.30±0.03	-0.14±0.03	F[1,29] = 19.26, p<0.001
Left posterior insular cortex	-39	-10	-3	239	4.15	0.11±0.03	-0.04±0.04	F[1,29] = 16.82, p<0.001
Left angular gyrus	-53	-70	20	1283	4.34	-0.22±0.02	-0.12±0.02	F[1,29] = 11.94, p=0.002
	-56	-54	41	1454	4.47	-0.20±0.02	-0.11±0.02	F[1,29] = 10.94, p=0.003
Middle cingulate cortex	1	-23	34	63	2.99	0.07±0.03	0.22±0.03	F[1,29] = 10.47, p=0.003
Left primary somatosensory cortex	-59	-16	40	93	2.94	0.12±0.02	0.04±0.02	F[1,29] = 8.62, p=0.006
Precuneus	6	-51	42	1314	4.21	-0.20±0.03	-0.08±0.03	F[1,29] = 15.50, p<0.001
Right primary somatosensory cortex	39	-21	49	255	3.02	0.45±0.03	0.36±0.03	F[1,29] = 8.38, p=0.007
Right dorsolateral prefrontal cortex	35	21	50	489	3.81	-0.05±0.02	0.05±0.03	F[1,29] = 8.75, p=0.005
Left dorsolateral prefrontal cortex	-30	28	53	60	3.39	0.05±0.03	0.14±0.03	F[1,29] = 7.01, p=0.013
Left posterior parietal cortex	-45	-58	57	221	4.08	-0.02±0.03	0.10±0.03	F[1,29] = 7.32, p=0.011

Supplementary Table 8. Location, level of significance, and cluster size of regions in consistent placebo analgesia responders where main effects of placebo were identified within the volume of regions showing main effect changes across all participants. Post-hoc three-way ANOVA determined whether functional changes were selective to placebo consistent responders, and an interaction between group (consistent responder, variable response, or consistent non-responder) and cream (control and XK50.113 placebo) over time was present. * Indicates post-hoc significance at p<0.05. Co-ordinates are in Montreal Neurological Institute (MNI) space.

	**MNI co-ordinates [x y z]**			**cluster size**	**Z-score**	**ANOVA consistent responders**	**3-Way ANOVA response x cream**
placebo>control							
Right cerebellar crus II	45	-70	-47	132	2.99	F[1,13]=7.87, p=0.01	F[1,28]=2.24, p=0.15
Left cerebellar crus I	-17	-87	-31	117	3.29	F[1,13]=11.86, p=0.004	F[1,28]=1.78, p=0.19
	-19	-86	-27	57	2.86	F[1,13]=5.31, p=0.03	F[1,28]=0.58, p=0.45
Left posterior orbitofrontal cortex	-24	8	-20	318	3.88	F[1,13]=9.13, p=0.01	F[1,28]=3.46, p=0.07
Right orbitofrontal cortex	16	46	-18	275	4.03	F[1,13]=14.90, p=0.002	F[1,28]=9.04, p=0.006*
Left middle frontal gyrus	-39	56	-14	70	3.19	F[1,13]=9.74, p=0.008	F[1,28]=2.52, p=0.12
Right middle frontal gyrus	31	45	-8	34	3.84	F[1,13]=10.92, p=0.006	F[1,28]=6.30, p=0.02*
Middle cingulate cortex	1	-23	33	30	2.97	F[1,3]=7.81, p=0.01	F[1,28]=1.89, p=0.18
Left angular gyrus	-59	-56	42	469	3.56	F[1,13]=7.39, p=0.02	F[1,28]=0.93, p=0.34
Precuneus	6	-51	43	256	3.29	F[1,13]=9.43, p=0.009	F[1,28]=2.24, p=0.14
Right dorsolateral prefrontal cortex	27	43	41	54	2.92	F[1,13]=12.96, p=0.003	F[1,28]=5.54, p=0.02*
	25	29	52	27	2.81	F[1,13]=10.88, p=0.006	F[1,28]=5.61, p=0.02*
Left dorsolateral prefrontal cortex	-29	27	54	86	2.98	F[1,13]=9.50, p=0.009	F[1,28]=6.59, p=0.01*
Left posterior parietal cortex	-45	-59	56	103	3.10	F[1,13]=9.75, p=0.008	F[1,28]=3.93, p=0.06
placebo<control							
Left posterior insula cortex	-40	-14	-4	179	3.57	F[1,13]=11.36, p=0.005	F[1,28]=8.42, p=0.008*
Right primary somatosensory cortex	41	-20	47	198	3.46	F[1,13]=15.31, p=0.002	F[1,28]=6.04, p=0.02*

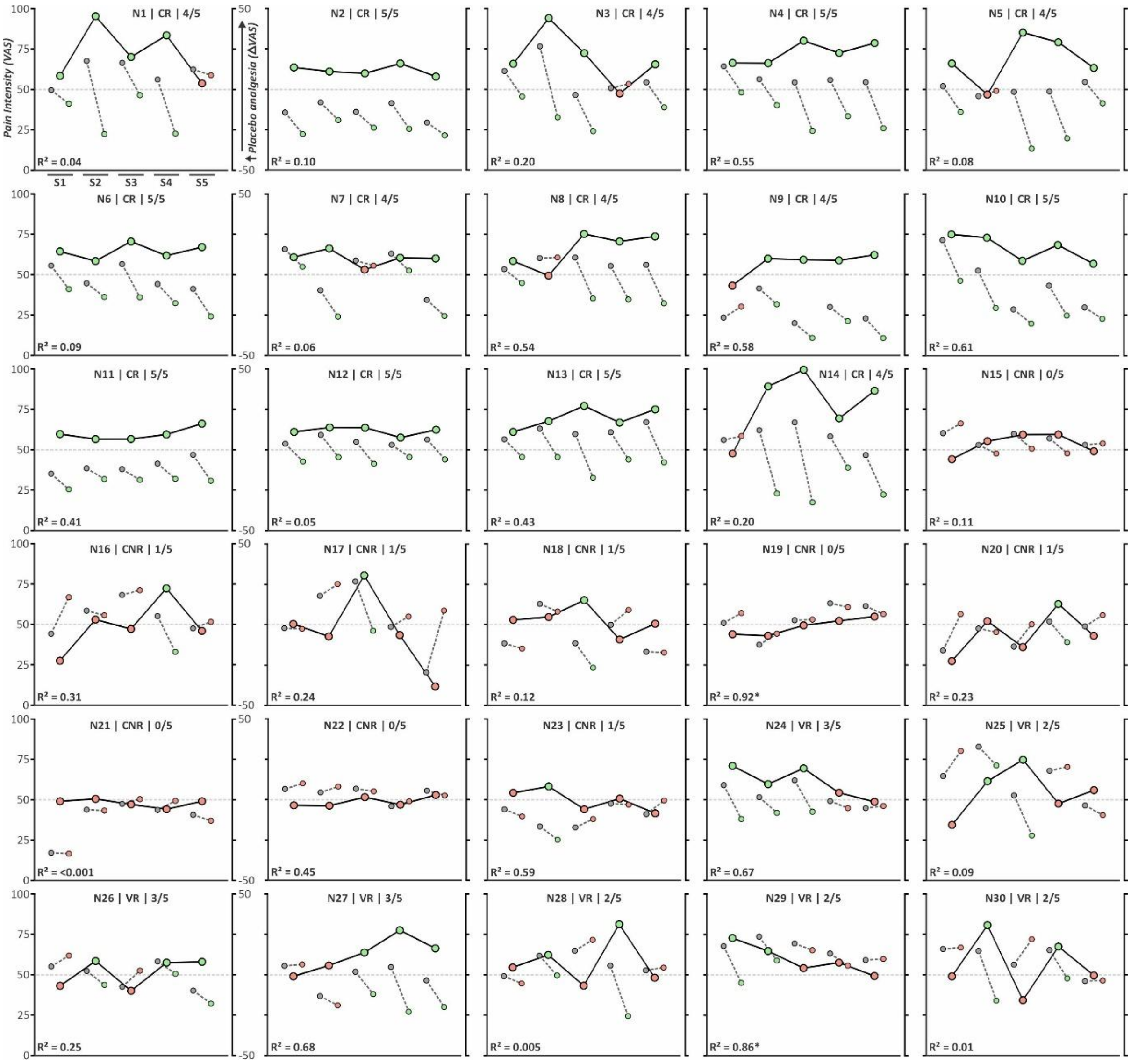

Supplementary Figure 1. Individual pain intensity responses to control and placebo XK50.113 cream sites during each session. N1 to N14 were characterized as consistent placebo responders, N15 to N23 consistent non-responders, and N24 to N30 variable responders. Y-axis (left) indicates mean pain intensity to each cream site, and Y-axis (right) shows pain intensity change during each session between creams. Grey circles indicate mean VAS to control site stimulation and green or red circles VAS to placebo cream stimulation, with green a significant placebo response and red a non-response. Doted lines join pain intensity ratings during control and placebo cream stimulations, whereas solid lines join changes in pain intensity (placebo-control). R^2 values indicate the relationship between changes in pain intensity (placebo-control) over time. Note that only in N19 consistent non-responder) and N29 (variable responder) was a significant linear association identified.

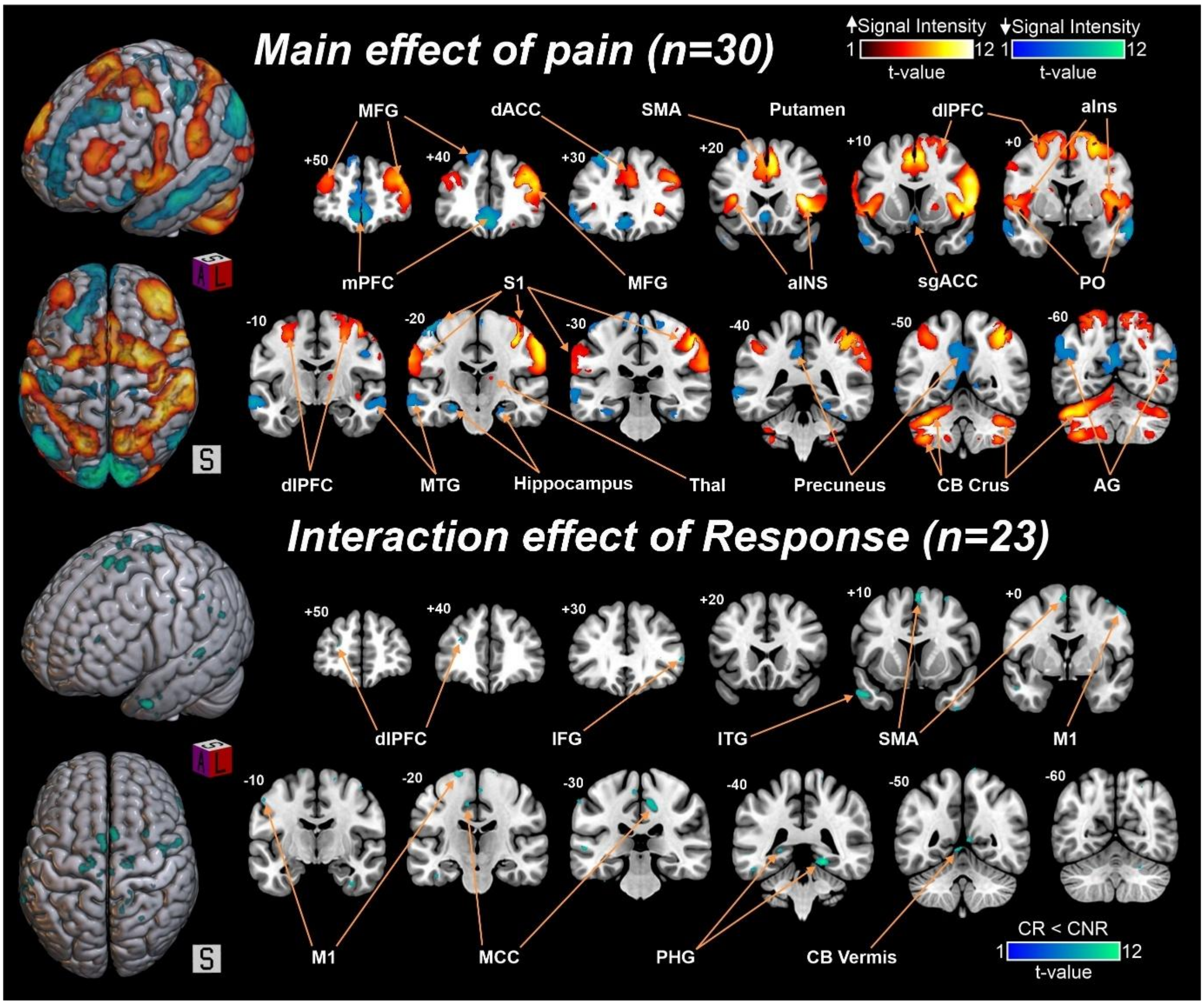

Supplementary Figure 2. Main effects of pain, and interaction effect response whole brain contrast maps. Each participant's first level contrast image for heat pain applied to the control cream site was entered into a factorial analysis with time entered as a five-layer factor to isolate cortical regions responsive to acutely applied thermal pain. Additionally, a 2x2x5 factorial analysis was generated with "response" and "cream site" loaded as separate two-layer factors, and "time" entered as a five-layer factor to generate interaction contrasts of cream site by response, isolating regions showing response-dependent opposing differences in activity between consistent responder and non-responder groups. Main effects of pain contrast images were thresholded at a family-wise error corrected rate of $p<0.05$, and the interaction effect of response contrast was thresholded at an uncorrected p-value of $p<0.001$, with a cluster forming threshold of 20 contiguous voxels. Top-down and left-facing 3D renders are provided on the left side of each image row, with navigation cubes orientating the spin applied to each render. Co-ordinates in the upper left of each coronal slice denote anatomical location in Montreal Neurological Institute (MNI) space. *MFG = mid frontal gyrus; dACC = dorsal anterior cingulate cortex; SMA = supplementary motor area; dlPFC = dorsolateral prefrontal cortex; aIns = anterior insula cortex; mPFC = medial prefrontal cortex; S1 = primary*

somatosensory cortex; sgACC = subgenual anterior cingulate cortex; PO = parietal operculum; MTG = middle temporal gyrus; thal = thalamus; CB Crus = cerebellar crus; AG = angular gyrus; IFG = inferior frontal gyrus; ITG = inferior temporal gyrus; M1 = primary motor cortex; MCC = mid cingulate cortex; PHG = parahippocampal gyrusl CB Vermis = cerebellar vermis.
