# Supplementary material for: Placebo analgesia persistence: evaluating behavioural stability in repeated trials
Source: Commun Biol. 2026 May 28;9:1133. doi: 10.1038/s42003-026-10377-8 (PMC13500666; doi:10.1038/s42003-026-10377-8)
Supplement: Supplementary file 3 [file 42003_2026_10377_MOESM3_ESM.pdf]

Description of Additional Supplementary Files

File name- Supplementary Data

Description - Raw and coded behavioural, psychological, and both main effect and responderspecific effect functional imaging cluster data supporting these findings can be found in the associated Supplementary Data excel document.
