# Supplementary material for: Placebo analgesia persistence: evaluating behavioural stability in repeated trials
Source: Commun Biol. 2026 May 28;9:1133. doi: 10.1038/s42003-026-10377-8 (PMC13500666; doi:10.1038/s42003-026-10377-8)
Supplement: Supplementary file 4 [file 42003_2026_10377_MOESM5_ESM.pdf]

nature portfolio

Corresponding author(s): Luke Anthony Henderson

Last updated by author(s): May 8, 2026

Reporting Summary

Nature Portfolio wishes to improve the reproducibility of the work that we publish. This form provides structure for consistency and transparency in reporting. For further information on Nature Portfolio policies, see our Editorial Policies and the Editorial Policy Checklist.

Statistics

For all statistical analyses, confirm that the following items are present in the figure legend, table legend, main text, or Methods section.

n/a	Confirmed	
☐	☒	The exact sample size (*n*) for each experimental group/condition, given as a discrete number and unit of measurement
☐	☒	A statement on whether measurements were taken from distinct samples or whether the same sample was measured repeatedly
☐	☒	The statistical test(s) used AND whether they are one- or two-sided<br>*Only common tests should be described solely by name; describe more complex techniques in the Methods section.*
☐	☒	A description of all covariates tested
☐	☒	A description of any assumptions or corrections, such as tests of normality and adjustment for multiple comparisons
☐	☒	A full description of the statistical parameters including central tendency (e.g. means) or other basic estimates (e.g. regression coefficient) AND variation (e.g. standard deviation) or associated estimates of uncertainty (e.g. confidence intervals)
☐	☒	For null hypothesis testing, the test statistic (e.g. *F*, *t*, *r*) with confidence intervals, effect sizes, degrees of freedom and *P* value noted<br>*Give P values as exact values whenever suitable.*
☒	☐	For Bayesian analysis, information on the choice of priors and Markov chain Monte Carlo settings
☒	☐	For hierarchical and complex designs, identification of the appropriate level for tests and full reporting of outcomes
☐	☒	Estimates of effect sizes (e.g. Cohen's *d*, Pearson's *r*), indicating how they were calculated

Our web collection on statistics for biologists contains articles on many of the points above.

Software and code

Policy information about availability of computer code

Data collection	Brain images were acquired at 3-Tesla using a 32-channel digital head coil (Ingenia CX, Philips, Best, The Netherlands). Participants were positioned supine with their head in the coil and sponges placed either side to support the head laterally and minimize motion. A T1-weighted anatomical image set covering the whole brain was collected as part of the first experimental session (repetition time = 7.2ms, echo time = 3.4ms, raw voxel size = 1.0x1.0x1.0mm, 170 sagittal slices, scan time = 5 mins). During each experimental visit two functional MRI sequences were also collected - one with the thermode positioned over the control cream site and the other with the thermode positioned over the XK50.113 placebo cream site. Each sequence consisted of 6 dummy and 246 dynamic gradient echo echo-planar measurements using blood oxygen level dependent (BOLD) contrast covering the entire brain. Images were acquired in an interleaved collection pattern with a multiband factor of 3 (repetition time = 1500ms, echo time = 30ms, raw voxel size = 2.0x2.0x2.0mm, 75 axial slices, scan time = 6min18sec).
Data analysis	Brain imaging data were analyzed in Matlab Version 2023a hosting Statistical Parametric Mapping version 12.<br>Predictive models were constructed in the Matlab Classification Learner Toolbox<br>Psychological, Behavioural, and Demographic data were analysed and all significance testing conducted within Graphpad Prism version 10.1.2 within a Windows operating system.

For manuscripts utilizing custom algorithms or software that are central to the research but not yet described in published literature, software must be made available to editors and reviewers. We strongly encourage code deposition in a community repository (e.g. GitHub). See the Nature Portfolio guidelines for submitting code & software for further information.

April 2023

Data

Policy information about availability of data

All manuscripts must include a data availability statement. This statement should provide the following information, where applicable:

- Accession codes, unique identifiers, or web links for publicly available datasets
- A description of any restrictions on data availability
- For clinical datasets or third party data, please ensure that the statement adheres to our policy

Raw and coded behavioural, psychological, and both main effect and responder-specific effect functional imaging cluster data supporting these findings can be found in the associated source document. Further data are available from the corresponding author (LAH), upon reasonable request

Research involving human participants, their data, or biological material

Policy information about studies with human participants or human data. See also policy information about sex, gender (identity/presentation), and sexual orientation and race, ethnicity and racism.

Reporting on sex and gender	The term sex assigned at birth is used throughout the manuscript and was collected both on a demographic questionnaire, and on participant completion of MRI safety forms - in all instances across the 30 participants of this investigation responses to each of these two questionnaires were consistent, indicating sex and gender were aligned in each of our participants.<br>Sex/gender was determined through self-reporting<br>Disaggregated sex and gender data is supplied in supplementary table 1, and consent was obtained to produce this data deidentified<br>for the purpose of publication by all participants.<br>Main effect of sex analysis on placebo responsivity were performed and are provided in supplementary table 2, to investigate whether an interaction between sex and consistency of placebo analgesia responses were present.
Reporting on race, ethnicity, or other socially relevant groupings	We do not report on any socially-constructed or socially-relevant categorization of participants in this investigation. The term "young" is used, since the mean age and age range of participants fell below 25 years.<br>The main effect of age was tested between response categories to ensure no confound of age was present between groups of participants that displayed altered consistency in evoking placebo analgesia responses. This analysis is provided in supplementary table 2
Population characteristics	All participants reported no current Chronic pain and 1 participant reported a history with chronic pain who was classified as a variable placebo responder and showed no outlier data either psychological nor in brain imaging which warranted removal from the participant cohort.
Recruitment	Participants were recruited through flyers placed at the imaging facility and the neighbouring university campus. Our sample was biased toward university-age students due to requiring through the week 9am-5pm availability for 5 repeated sessions over 8 weeks each lasting roughly 1.5 hours to complete the study. We have noted this limitation of narrow sample population within the manuscript text lines 369-374.
Ethics oversight	University of Sydney Human Research Ethics Committee Stream 3 - approval code HREC 2022/590

Note that full information on the approval of the study protocol must also be provided in the manuscript.

Field-specific reporting

Please select the one below that is the best fit for your research. If you are not sure, read the appropriate sections before making your selection.

☒ Life sciences ☐ Behavioural & social sciences ☐ Ecological, evolutionary & environmental sciences

For a reference copy of the document with all sections, see nature.com/documents/nr-reporting-summary-flat.pdf

Life sciences study design

All studies must disclose on these points even when the disclosure is negative.

Sample size	Our sample comprised 34 pain-free healthy control participants (18 Female, mean age = 21.59±0.44 years [±SEM]; range 19-31 years)<br>An a priori power analysis using results from a previous behavioural study investigating behavioural consistency of placebo analgesia revealed a sample size of 21 would be necessary to detect similar effects with 90% power ($d = 0.67$, $\alpha = 0.05$, power = 0.9). Furthermore, power analysis using NeuroPower indicated that our total sample size of 30 participants provides 83% power under Bonferroni correction (α=0.05).
Data exclusions	Four participants were excluded from final analyses due to three failing to return after the first or second experimental sessions, and the other with dental implants which irrevocably distorted functional brain imaging data.
Replication	The nature of the experimental design being longitudinal meant that the same study procedures were replicated in five seperate sessions across an eight-week period, each seperated by a two-week interval. The ratio of significant analgesic responses within the cohort did not significantly differ between sessions, suggesting this phenomena to be stable and replicable in the majority of participants.

Randomization	The experimental design was within-subjects, as such at the time of completing the study no participants were randomized, and all participants completed all experimental procedures in a fixed-order. Participants were allocated one of three response groups based on pain rating data generated during the test phase, with interactions of psychology, demography, and neural activation determined between groups.
Blinding	Experimenters were not blinded to which cream was neutral and placebo as response conditioning required deceptive application of reduced noxious input to the placebo cream specifically during the conditioning phase. The placement of the neutral and placebo creams were counterbalanced between participants and within-participants between sessions to reduce effects of ordering and sensitivity

Reporting for specific materials, systems and methods

We require information from authors about some types of materials, experimental systems and methods used in many studies. Here, indicate whether each material, system or method listed is relevant to your study. If you are not sure if a list item applies to your research, read the appropriate section before selecting a response.

Materials & experimental systems

n/a	Involved in the study
☒	☐ Antibodies
☒	☐ Eukaryotic cell lines
☒	☐ Palaeontology and archaeology
☒	☐ Animals and other organisms
☒	☐ Clinical data
☒	☐ Dual use research of concern
☒	☐ Plants

Methods

n/a	Involved in the study
☒	☐ ChIP-seq
☒	☐ Flow cytometry
☐	☒ MRI-based neuroimaging

Plants

Seed stocks	This section was ticked as "N/A" above but has still populated in this document - only MRI-based neuroimaging from the above list is relevant to this study
Novel plant genotypes	This section was ticked as "N/A" above but has still populated in this document - only MRI-based neuroimaging from the above list is relevant to this study
Authentication	This section was ticked as "N/A" above but has still populated in this document - only MRI-based neuroimaging from the above list is relevant to this study

Magnetic resonance imaging

Experimental design

Design type	Event-related design
Design specifications	10 events of acute noxious thermal input per cream site (2 cream sites), for a total of 20 events per session. Each participant underwent 5 sessions seperated by 2-week intervals, for a total of 100 events throughout the duration of the study.<br>Each event lasted 12-seconds, and was seperated by a 12-second inter-trial interval.
Behavioral performance measures	A continuous recording of perceived pain intensity was recorded from participants both during response conditioning and while within the MRI scanner - participants controlled the position of a slider in both settings using their left index and middle finger to indicate their currently perceived pain between 0 - "no pain" and 100 - "worst imaginable pain". Paired t-tests were used to characterize behaviour within a group, aswell as bootstrapped permutation to classify a participant during each session as showing a placebo analgesia response. This procedure is described in manuscript lines 459-461.

Acquisition

Imaging type(s)	Structural and Functional
Field strength	3-Tesla
Sequence & imaging parameters	T1-weighted anatomical image set covering the whole brain was collected as part of the first experimental session (repetition time = 7.2ms, echo time = 3.4ms, raw voxel size = 1.0x1.0x1.0mm, 170 sagittal slices, scan time = 5 mins). Each fMRI sequence consisted of 6 dummy and 246 dynamic gradient echo echo-planar measurements using blood

oxygen level dependent (BOLD) contrast covering the entire brain. Images were acquired in an interleaved collection pattern with a multiband factor of 3 (repetition time = 1500ms, echo time = 30ms, raw voxel size = 2.0x2.0x2.0mm, 75 axial slices, scan time = 6min18sec)

Area of acquisition: Whole brain images were recorded in both structural and functional image series

Diffusion MRI ☐ Used ☒ Not used

Preprocessing

Preprocessing software: Statistical Parametric Mapping version 12, Computational Anatomy Toolbox version 12.
Functional images were slice-timing and motion-corrected with the parameters visually inspected for volume-to-volume motion exceeding 1mm in rotational or 0.5 degree in translational planes. No participant displayed exaggerated motion in any of the five sessions and as such all image series remained included for further analyses. Images were then linearly detrended to remove global signal drift, passed through the DRIFTER toolbox to remove physiological noise relating to cardiac (60-120 beats per minute +1 harmonic) and respiratory (8-25 breaths per minute +1 harmonic) frequency, and noise relating to volume-to-volume movement removed by attached the motion parameter files as regressors following a linear modelling of realignment parameters (LMRP) procedure. Each participants' fMRI image series from the five sessions were then resliced into 1.0mm isotropic voxels and co-registered to the corresponding T1-anatomical image collected in the first session. The T1 image was then normalized to the Montreal Neurological Institute (MNI) template and these parameters applied to each of the functional image series. Normalized functional image series were finally spatially smoothed using a 6mm full-width at half maximum Gaussian filter.

Normalization: All data were normalized to the Montreal Neurological Institute (NMI) template using a standard nonlinear affine transformation procedure built within SPM12 in Matlab.

Normalization template: MNI ICBM152 - default template of the Computational Anatomy Toolbox

Noise and artifact removal: Temporal noise - Slice timing correction using SPM12 standard package
Global signal noise - linear detrending using SPM12 standard packages
Physiological noise - modelled and removed using the DRIFTER toolbox packaged in SPM12
Motion artefact - motion parameters calculated from raw data series were added as covariates and regressed out using a linear modelling of realignment parameters procedure

Volume censoring: The first 5 dynamic volume of each sequence were sensored due to scanner signal saturation. The baseline period was >70 volumes before the first event occurred, so this censoring has little to no impact on event-related signal

Statistical modeling & inference

Model type and settings: Random-effects analyses were conducted at the first level to determine voxel signal changes dependent on the event-related model (noxious stimuli). Main effect of scan (control versus placebo) over time analysis was first conducted, before sub analyses of interaction by group allocation were conducted based on the number of sessions individuals mounted significant placebo analgesia responses. All second level analyses were conducted as full factorial analyses - with either 1, 2, or 3 factors entered. Figure 3 represents the main effect of placebo analysis. Figure 4 was constructed in a two factor full factorial analysis with "scan" and "time" entered as two and five
layer factors, respectively. Supplementary figure 2 includes analyses from a one factor factorial analysis with only "time" entered as a five layer factor, and a three factor analysis with "response", "scan", and "time" entered as two, two, and five layer factors, respectively.

Effect(s) tested: Main effects of scan were constructed in consistent placebo responders, with values extracted and split into each session (time) period to plot changes in whole brain regions responsive to evoking placebo analgesia irrespective of any specific session. Interaction terms of response x cream were computed to determine whether these circuits showed significant differences between consistent placebo responders and consistent placebo non-responders and are summarized in table 4.

Specify type of analysis: ☒ Whole brain ☐ ROI-based ☐ Both

Statistic type for inference: voxel-wise

(See Eklund et al. 2016)

Correction: p<0.001 uncorrected for multiple comparisons with a cluster forming threshold of 50 contiguous voxels, small-volume correction applied to each resulting cluster to reduce type II error likelihood.

Models & analysis

n/a	Involved in the study
☒	☐ Functional and/or effective connectivity
☒	☐ Graph analysis
☒	☐ Multivariate modeling or predictive analysis
